# Molecular evaluation of orphan Afghan common wheat (*Triticum aestivum* L.) landraces collected by Dr. Kihara using single nucleotide polymorphic markers

**DOI:** 10.1186/s12870-014-0320-5

**Published:** 2014-11-29

**Authors:** Alagu Manickavelu, Abdulqader Jighly, Tomohiro Ban

**Affiliations:** Kihara Institute for Biological Research, Yokohama City University, Yokohama, 244-0813 Japan; International Centre for Agricultural Research in the Dry Areas (ICARDA), P. O. Box 5466, Aleppo, Syria

**Keywords:** Afghan wheat landraces, Botanical varieties, Genetic diversity, Population structure, Single nucleotide polymorphism

## Abstract

**Background:**

Landraces are an important source of genetic diversity in common wheat, but archival collections of Afghan wheat landraces remain poorly characterised. The recent development of array based marker systems, particularly single nucleotide polymorphism (SNP) markers, provide an excellent tool for examining the genetic diversity of local populations. Here we used SNP analysis to demonstrate the importance of Afghan wheat landraces and found tremendous genetic diversity and province-specific characteristics unique to this geographic region.

**Results:**

A total of 446 Afghan wheat landraces were analysed using genotype by sequencing (GBS) arrays containing ~10 K unique markers. Pair-wise genetic distance analyses revealed significant genetic distances between landraces, particularly among those collected from distanced provinces. From these analyses, we were able to divide the landraces into 14 major classes, with the greatest degree of diversity evident among landraces isolated from Badakhshan province. Population-based analyses revealed an additional 15 sub-populations within our germplasm, and significant correlations were evident in both the provincial and botanical varieties. Genetic distance analysis was used to identify differences among provinces, with the strongest correlations seen between landraces from Herat and Ghor province, followed closely by those between Badakhshan and Takhar provinces. This result closely resembles existing agro-climatic zones within Afghanistan, as well as the wheat varieties commonly cultivated within these regions. Molecular variance analysis showed a higher proportion of intra-province variation among landraces compared with variation among all landraces as a whole.

**Conclusion:**

The SNP analyses presented here highlight the importance and genetic diversity of Afghan wheat landraces. Furthermore, these data strongly refute a previous analysis that suggested low genetic diverse within this germplasm. Ongoing analyses include phenotypic characterisation of these landraces to identify functional traits associated with individual genotypes. Taken together, these analyses can be used to help improve wheat cultivation in Afghanistan, while providing insights into the evolution and selective pressures underlying these distinct landraces.

**Electronic supplementary material:**

The online version of this article (doi:10.1186/s12870-014-0320-5) contains supplementary material, which is available to authorized users.

## Background

Wheat (*Triticum aestivum*) is the third most important cereal crop worldwide in terms of production and the most important in terms of calorie consumption, with overall production increasing year after year [1]. However, in developing countries such as Afghanistan, wheat production has declined steadily, an alarming trend in countries already struggling to meet basic food demands. In order to achieve sustainable production goals, most national programs have begun either exploiting existing natural diversity to identify strains suitable for specific regions or climates or have simply used elite varieties developed by private or international agricultural research centres. Regardless of the approach taken, identifying important alleles and other genetic information present in existing gene pools will be necessary to achieve optimal crop yields. Moreover, the establishment of self-driven germplasm activities is more sustainable, as this approach utilises native landraces, which are well suited to local environments.

Landraces have been identified as distinct, locally-adapted species with a high capacity to tolerate biotic and abiotic stresses, resulting in higher sustainable yields, as well as intermediate yields under low input agricultural conditions [2]. Populations such as these arose as a result of both natural and artificial selection, adapting not only to crop centres of origin, but also to new environments following transplantation.

Afghanistan is the third largest centre of origin for domesticated crops worldwide [3], having played an important role in the domestication of wheat, barley (*Hordeum vulgare*), chickpeas (*Cicer arietinum*), peas (*Pisum sativum*), and rye (*Secale cereale*). However, frequent armed conflicts and other factors have led this country to lose all known germplasm collections developed to date. Fortunately, one long-running scientific expedition led by Dr. Hitoshi Kihara and others between 1950 and 1970 established an extensive Afghan wheat landraces collection, which is now housed in Japan. While other Afghan wheat collections do exist, the collection of landraces found in the Kihara Institute for Biological Research, Japan is thought to be unique in terms of the number of sites visited, the diversity of their environmental conditions, and the overall number of landraces collected [4]. Moreover, in contrast to other landraces, those of this collection are thought to be homozygous, since they were allowed to propagate by self-pollination over the course of several generations of genotypic studies. The genetic diversity contained within may therefore hold significant potential for both Afghanistan and beyond; however, significant work is needed to characterize these samples fully.

The recent development of molecular markers and high throughput systems has revealed a wealth of genotypic information for a wide variety of crops and plants [5,6]. Among these, single nucleotide polymorphisms (SNPs) are the most common type of sequence variation in the genome [7], making them well suited for genomics approaches requiring a high number of markers, such as association mapping [8] and genomic selection [9]. High-throughput SNP genotyping platforms have long been available for diploid crops such as maize [10] and barley [11], and SNP arrays were developed recently for wheat [12,13]. SNP analysis has been used successfully to characterize rice landraces [14]; however, similar work in other landrace collections, such as wheat, has been minimal [15]. Here we examined a large, yet poorly characterized wheat landrace collection from Afghanistan to determine the genetic diversity, population structure, and other characteristics associated with genetic polymorphisms.

## Results and discussion

### The Kihara Afghan wheat landrace (KAWLR) collection and its importance

**Figure 1:**
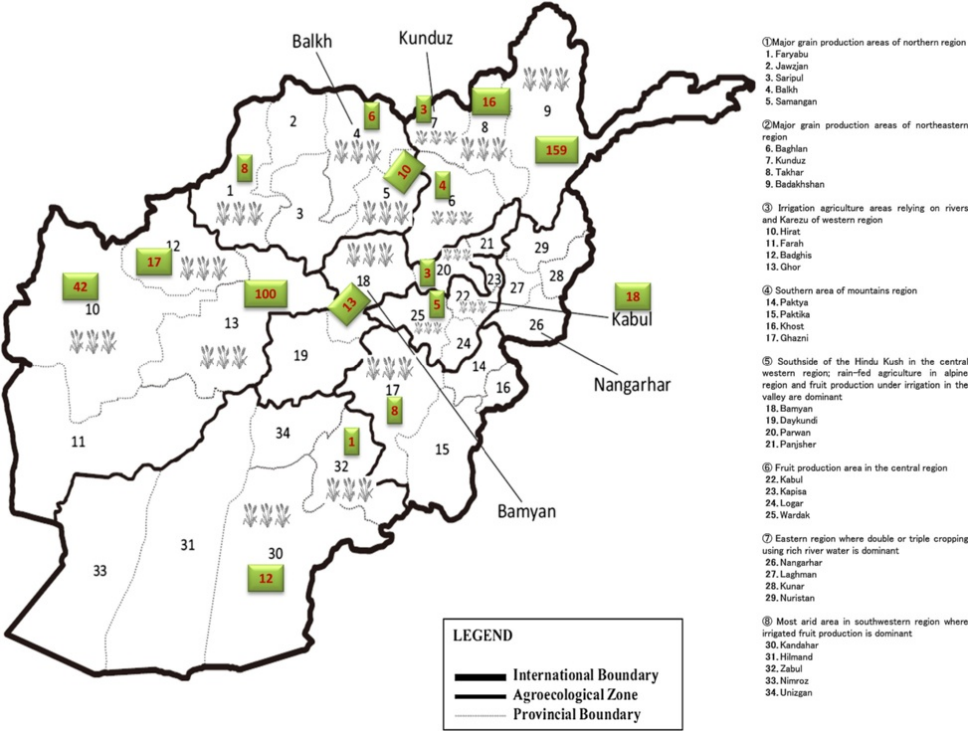
**Geographical location of Afghan wheat landraces and their grouping based on agro-ecological zones.** The map is divide into eight agro-ecological zones according to FAO [Food and Agricultural Organization]. The number of accessions from each province are shown in green squared boxes.

Although the importance of landraces in terms of both conservation and utilisation remain controversial [2], much of this uncertainty stems from the lack of reliable data regarding the use and implementation of these resources [16-18]. Over the past few decades, significant efforts have been invested in the collection, preservation, and use of landraces worldwide. However, these efforts have failed to address the role of Afghan wheat landraces, a significant absence given the historical significance of this region in the domestication of wheat. While little remains of the local Afghan stocks, private collections, such as the one initiated by Dr. Kihara, have preserved much of the original diversity, accounting for ~500 unique Afghan landraces [19]. Furthermore, the Kihara collection was maintained and preserved in both pure and homozygous states, increasing the novelty of these materials relative to other landraces. In addition, this germplasm contains representative landraces from all of the wheat-growing areas of Afghanistan, across eight agro-climatic zones, allowing for the most comprehensive study of the Afghan wheat gene pool to date (Figure 1) [1]. Recently Mitrofanova et al. [16] examined the genetic diversity of Afghan bread wheat landraces by compiling data available through all of the major gene banks worldwide. However, the scope of this analysis included only a small subset of available lines, with characterizations limited to just a handful of SSR markers.

**Figure 2:**
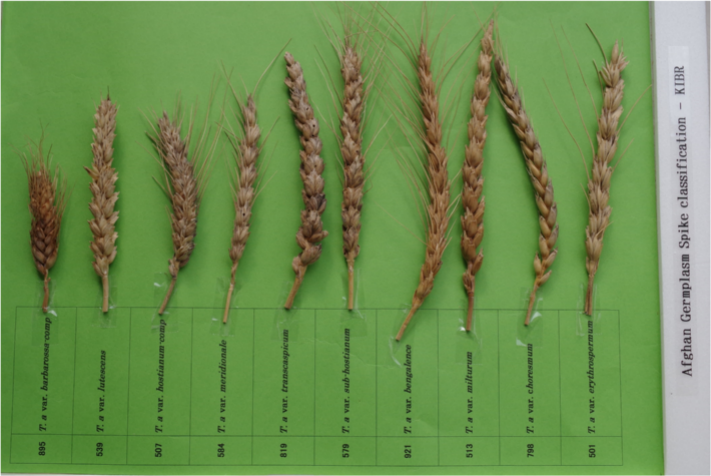
**Classification of spikes in Afghan wheat landraces.** The germplasm number and botanical variety for each landrace are mentioned in the attached label. Although a total of 19 botanical varieties were identified, only those showing clear variation are shown here.

In addition to genetic variations, phenotypic descriptions of this germplasm were also investigated, resulting in a total of 47 distinct botanical varieties. A previous study by Buerkert et al. [17] identified 19 botanical varieties in Afghan wheat landraces, although the number of unique landraces included in that study was significantly smaller than in the collection described here. Variations in spike morphology were also explored, revealing 10 different spike types, ranging from var. *compactum* (Alef.) Velican to var. *speltoides* (Alef.) Velican (Figure 2). Such an abundance of both genetic and phenotypic diversity evident in these materials makes this collection an essential resource for rebuilding the Afghan wheat industry and for improving the diversity of the Afghan wheat germplasm. Outside of Afghanistan, this germplasm represents an important resource for understanding wheat genetics and for developing new strains that may be better adapted to local climates.

### Analysis of SNP markers

**Figure 3:**
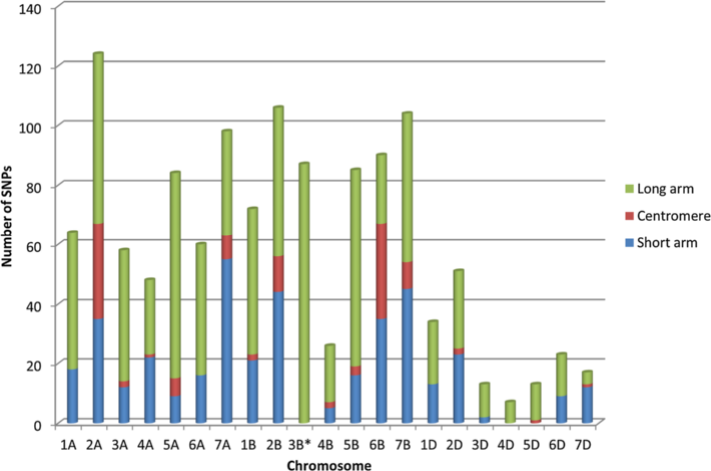
**Chromosomal locations of SNP markers.** Markers were arranged along the long arm (green), centromere (dark red), and short arm (blue), respectively. For the markers on chromosome 3B, there are no details regarding chromosome arm.

Following GBS analysis, data were filtered to remove SNPs exhibiting a minor allele frequency ≤10%, resulting in a total of 8969 SNP markers. Of these markers, 2770 were identified as transition markers, while 1738 represented transversion SNPs. Chromosomal alignments were successful for 1264 markers, with SNPs distributed across all 21 chromosomes (Figure 3). The highest number of markers was found on chromosome 2A and the lowest in chromosome 4D; a majority of markers were located in close proximity to the centromeres. As expected, more markers were identified in the A and B genomes than in the D genome, consistent with a previous study [20], indicating a need for targeted marker development for the D genome. Preliminary efforts to address this deficiency include the development of a DArT marker array based on 81 *Aegilops tauschii* Coss accessions [21], although the resulting marker coverage remains lower than desired.

### Individual genetic distances and kinship relationships

**Figure 4:**
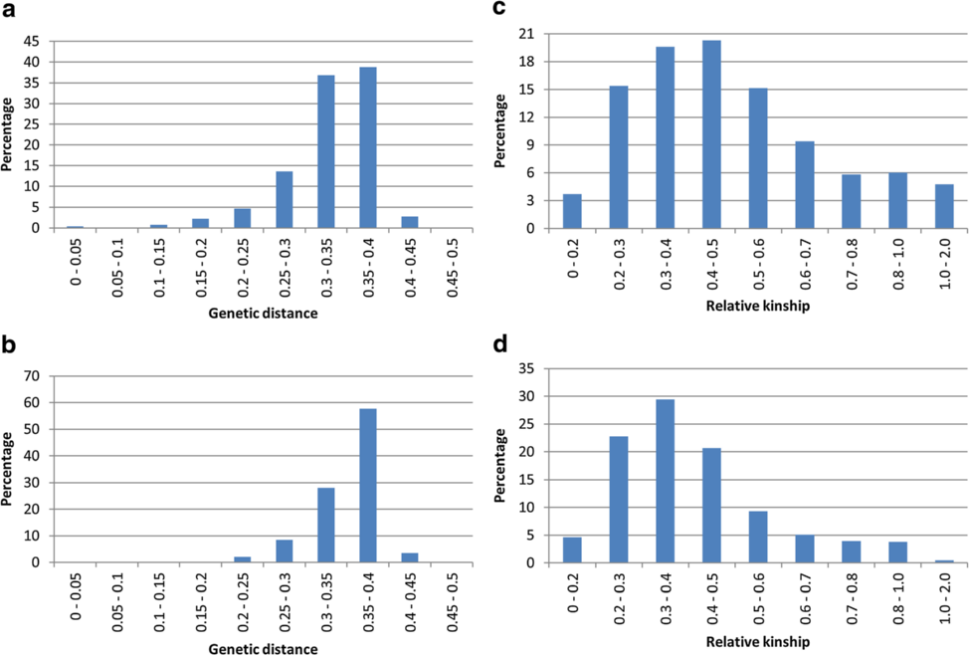
**Distribution of pairwise (a) Roger’s genetic distance among the landraces; (b) Roger’s genetic distance between landraces and controls; (c) relative kinship among landraces, and (d) relative kinship calculated between landraces and controls.**

The pairwise Roger genetic distance between each of the 446 landraces ranged from 0.002 to 0.47 with an overall mean distance of 0.33. While such a high degree of divergence is uncommon for a national collection of self-pollinated landraces, this result is not without precedent; Semagn et al. [22] reported a similar mean distance of ~0.35 for a diverse set of CIMMYT maize inbred lines. Of the 99,235 pairwise distances, 75,015 (75.6%) fell between 0.3 and 0.4 (Figure 4a), with only 400 (0.4%) exhibiting values <0.1. Comparisons between selected controls resulted in a total of 19,624 pairwise distances, of which 16,833 (85.8%) fell between 0.3 and 0.4, and only 22 (0.11%) exhibited distances <0.2 (Figure 4b). Taken together, these results are indicative of a very low degree of genetic redundancy within this collection.

In contrast to the high overall genetic distances between landraces, the relative kinship coefficients between pairs of samples ranged from 0 to 1.99, with an average value of 0.5, which is a bit higher than that seen in the CIMMYT maize study (0.37) [22]. The majority of the pairwise kinships (82,852; 83.5%) fell below 0.7 (Figure 4c), while 18,019 (91.8%) of the pairwise kinship coefficients between controls fell below 0.7 (Figure 4d). Only 4,695 (4.7%) of the pairwise kinship coefficients between landraces and 88 (0.45%) of the pairwise kinship coefficients between landraces and controls were >1.0, suggesting that the vast majority of landraces described in this study may be contributing new alleles to the Afghan gene pool, even though ~70% of them were collected from only three provinces (Herat, Ghor, and Badakhshan; Additional file 1).

### Dendrogram

**Figure 5:**
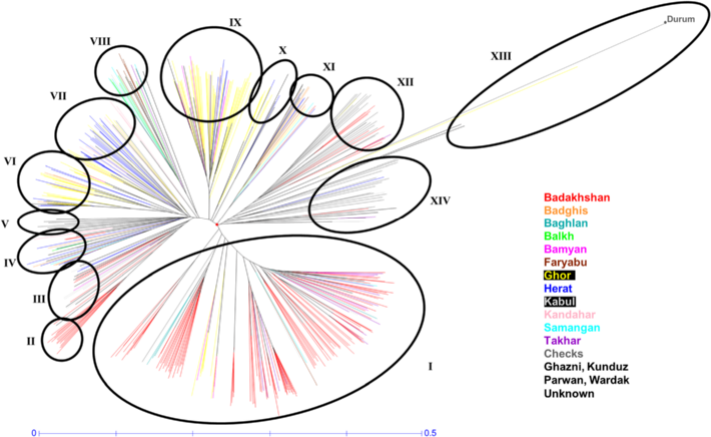
**Diversity of Afghan wheat landraces.** Each province is identified using a different colour. Landraces with unknown origins and those collected from Ghazni, Kunduz, Parwan, and Wardak provinces were left unshaded. Individual clade dendrograms are shown in Additional file 2: Figure S2.

High-throughput SNP arrays were used to evaluate 446 KAWLR samples and 45 controls originating from landraces of neighbouring countries, along with improved Afghan varieties, and one durum wheat genotype. Phylogenetic analysis revealed the complex nature of the diversity present in these landraces, which could be divided into 14 major clades (Figure 5, Additional file 2). Among these 14 clades, clade I accounted for ~30% of all landraces. This clade consisted of landraces collected from Badakhshan, Baghlan, Bamyan, and Takhar provinces, along with six landraces from Ghor, four from Kabul, two from Samangan, and one from Faryab. The provinces of Badakhshan, Baghlan, Bamyan, and Takhar are all located in the northeast region of Afghanistan, where their wheat landraces are expected to cluster together. More surprising was the inclusion of an Ishkashim landrace in this clade, considering its predecessor originated in Tajikistan. On the other hand, clade II was limited to landraces collected from Badakhshan province, with the exception of one landrace from Parwan. Landraces from the north central provinces of Balkh, Samangan, and Faryab clustered primarily within clade VIII. Clades VI and IX were comprised of landraces from Ghor, Herat, Wardak, Badghis, and Bamyan provinces, while clade X contained landraces collected from Ghor, Herat, and Wardak.

Clade VII contained the Iranian landraces NBRP47 and NBRP105, along with most of the Herat and Kandahar landraces, and an additional 11 landraces from Ghor. Geographically speaking, Ghor province is located in the centre of Afghanistan and is surrounded by the provinces of Herat, Wardak, Badghis, Kandahar, and Bamyan; this central location likely accounts for the overlap among these regions. Clades III, IV, and XI included a mixture of Afghan landraces belonging to different provinces, along with control NBRP48, which clustered in clade IV.

The remaining controls were grouped mainly into three clades: V, XII and XIV, of which clade V was comprised exclusively of controls. The rest of the hexaploid wheat controls, accounting for 30 genotypes, clustered within clades XII and XIV alongside 23 Afghan landraces (~5% of the total germplasm), consisting of 10 from Badakhshan, five from Kabul, two from Takhar, one from Kunduz, one from Ghor, one from Herat, and three from unknown sources. The significant divergence between the Afghan landraces and controls highlights the novelty of this collection and its potential value for the development of new wheat varieties.

As expected, the durum wheat genotype was clustered as an out-group in this analysis, although four Afghan landraces also clustered independently of other landraces in the highly differentiated clade XIII. Table 1 summarizes the distances and the relative kinship relationships within this clade and in relation to the entire germplasm. The durum genotype had the lowest kinship and the greatest distance relative to the germplasm mean; its highest kinship and shortest distance was with landrace 818. Of the Afghan landraces, three (743, 942, and 943) exhibited very high kinship and short genetic distances among each other; medium kinship relationships and distances were seen with landrace 818, while low kinship and long distances were seen in comparison to the durum genotype and germplasm means. Taken together, these three genotypes appear unique in relation to other clades, and may therefore harbour distinctive genes that could be exploited for breeding purposes. In contrast, landrace 818 appears to be a variety of durum wheat (*Triticum durum* Desf.), a distinction most likely the result of misclassification or human error.

**Table 1:** **The kinship coefficients (below diameter) and the distances (above diameter) among highly differentiated landraces; mean germplasm distances and the durum control are also shown**

**Landrace**	**743**	**818**	**942**	**943**	**Durum**	**Average**
743	-	0.32	0.21	0.004	0.39	0.43
818	0.53	-	0.33	0.32	0.25	0.36
942	1.05	0.52	-	0.21	0.39	0.43
943	1.98	0.53	1.03	-	0.39	0.44
Durum	0.23	0.88	0.23	0.26	-	0.45
Average	0.05	0.37	0.07	0.04	0.009	-

Full descriptions of each landrace number are available in Additional file 1: Table S1.

### Population structure

**Figure 6:**
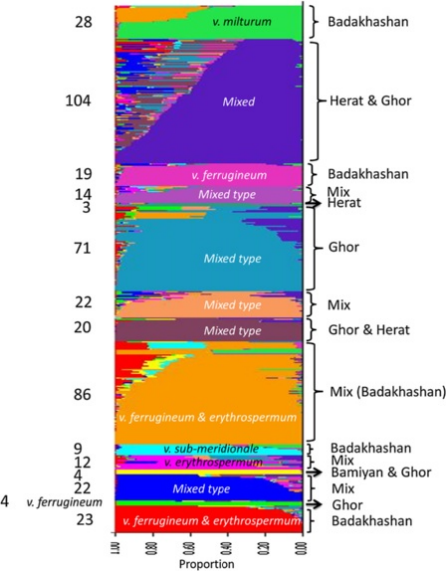
**Population structures of Afghan wheat landraces according to collection site and botanical variety.** Population structure analysis resulted in 15 sub-populations (K = 15). Details of the botanical variety composition are indicated for each group. Mixed type structure is defined as landraces lacking a specific botanical variety and those in which the province of origin is not known.

To further clarify our diversity analysis and to better estimate population subdivisions, a population structure analysis was performed using only KAWLR samples. Samples were analysed using STRUCTURE software [23], revealing 15 distinct sub-populations (K = 15) within our germplasm. In order to differentiate these sub-populations, samples were further categorised based on collection site, taxonomy, and morphology (Figure 6). The landraces of Badakhshan province alone were grouped into five different sub-populations, while the sub-population with the highest number of accessions (104 acc.) combined landraces collected from neighbouring Herat and Ghor provinces. When comparing sub-populations based upon botanical varieties, nine sub-populations could be identified as having unique botanical varieties. For instance, the var. *milturum* (Alef.) Velican from Badakhshan province was grouped exclusively with landraces collected from high elevations. Other examples include var. *ferrugineum* (Alef.) Velican and var. *erythrospermum* (Alef.) Velican, the major varieties found in our collection, which were often grouped together. Overall these results highlight the considerable diversity present in our germplasm, along with the ability of STRUCTURE analysis to better connect genomic diversity with its corresponding phenotypic outcomes, such as geographic distribution and botanical varieties.

The Badakhshan samples contained the greatest degree of diversity in our study, which can be used to adapt existing wheat strains to particular agro-climatic zones. Afghanistan is divided into eight agro-climatic zones [1], each of which is unique in terms of optimum crop yields. For wheat cultivation, Afghanistan is classified as mega environment 7, suitable for facultative wheat production with irrigation [24]. However, an FAO [Food and Agricultural Organization] report, combined with other project studies, showed that nearly 70% of Afghan land used for wheat production is within areas receiving sub-optimal rainfall, consistent with the need for selective breeding to adapt wheat varieties to local conditions.

Outside of botanical varieties, population structures were also affected strongly by the collection year. For instance, genotypes collected before 1979 were classified either primarily or completely within sub-populations 2–5, 8–11, and 14, whereas samples collected in 1979 or later grouped primarily in sub-populations 1, 6, 7, 12, 13, and 15. Moreover, landraces collected before 1979 were distributed across a higher number of sub-populations, with an average major sub-population contribution of 77.6%, compared with 84.7% for landraces collected after 1979. This analysis indicates a shift towards lower overall genetic diversity over time, with less deviation from a landrace’s major sub-population. This observation is consistent with our original assumptions, in that increasing wheat cultivation during this time period led to a reduction in overall genetic variation due to the greater availability and deployment of improved varieties, along with substantial changes in living conditions. Similar trends have continued in the years since this collection was completed, highlighting the need for alternative approaches to wheat cultivation in this region [25].

### Geographical sub-population analyses

**Figure 7:**
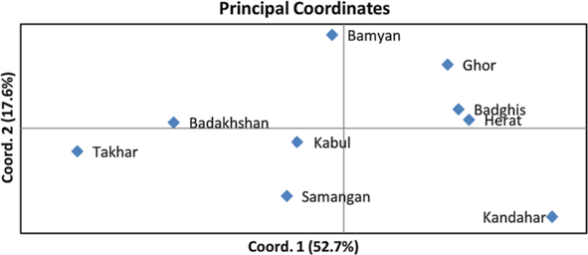
**Population structures of 385 Afghan wheat landraces collected from nine provinces (K = 2 to 10); only provinces with** ≥**10 landraces were used in this analysis.**

Next, landrace population structures were re-estimated using only 385 landraces collected from Badakhshan, Ghor, Herat, Takhar, Kabul, Badghis, Kandahar, Bamyan, and Samangan provinces, resulting in 10 distinct sub-populations. The strongest divisions were seen among landraces isolated from Badakhshan and Takhar provinces, as these two regions grouped independently of the remaining regions when divided into two groups (K = 2; Figure 7). Increasing the number of clusters resulted in division of the Badakhshan samples into seven sub-populations, of which five were unique to this province.

Nei genetic distances were also calculated using only the landraces described above. The most closely related sub-populations were from Herat and Ghor, followed by Badakhshan and Takhar, with genetic distances of 0.027 and 0.035, respectively. The largest distances were seen between Takhar landraces and those collected from Kandahar and Badghis (0.267 and 0.199, respectively; Table 2). The average Nei distance was the lowest (0.074) between all Kabul genotypes and those of other sub-populations, whereas Takhar and Kandahar genotypes were the most divergent (0.149 and 0.143, respectively). This result was somewhat surprising, given that Kabul genotypes showed the highest mean heterozygosity value (0.336), while the Kandahar genotypes exhibited the least amount (0.208).

**Table 2:** **Nei’s genetic distance of Afghan wheat landraces from selected provinces**

**Badakhshan**	**Badghis**	**Bamyan**	**Ghor**	**Herat**	**Kabul**	**Kandahar**	**Samangan**	
0.130								**Badghis**
0.075	0.092							**Bamyan**
0.105	0.054	0.036						**Ghor**
0.111	0.039	0.070	0.027					**Herat**
0.046	0.085	0.064	0.066	0.061				**Kabul**
0.197	0.109	0.149	0.090	0.064	0.122			**Kandahar**
0.074	0.093	0.102	0.101	0.088	0.056	0.142		**Samangan**
0.035	0.199	0.125	0.183	0.186	0.090	0.267	0.103	**Takhar**

**Figure 8:**
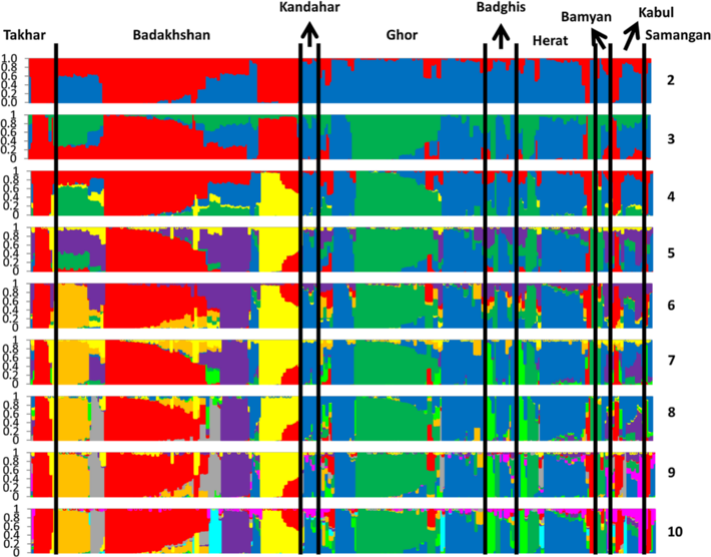
**Principal component analysis of Afghan wheat landraces.** The first coordinate explained 52.7% of the variability, while the second one accounted for an additional 17.6%.

The Nei genetic distance analyses were consistent with those of our PCA analysis, indicating a strong reproducibility across analytical methods (Table 2 and Figure 8). The first and the second principle coordinates account for over two-thirds of the total genetic variation among the nine sub-populations; the first coordinate accounted for 52.7% of the variability and the second for an additional 17.6%. Interestingly, the provinces of Badghis, Herat, and Ghor were clustered in both coordinates, while the provinces of Kabul, Samangan, and Bamyan were clustered close together on one coordinate but far apart on the second. Of the remaining provinces, Badakhshan and Takhar provinces clustered together tightly, while Kandahar failed to cluster with any of the other groups. These results are consistent with a previous study that examined the phylogeny and population structure of these regions.

Finally, an analysis of molecular variance (AMOVA) was performed under different conditions (Table 3). The resulting F-statistic indicated a significant proportion of variance among all cases (p <0.001), with a high degree of variance among varieties within a province (86%) but a low proportion of variance nationwide (14%; Table 3). Since landraces collected from Badakhshan and Takhar provinces clustered together so tightly when analysed using only two groups (K = 2; Figure 8), we performed a second round of AMOVA in which we considered these landraces as either one population or as one region consisting of two distinct populations, with all remaining samples clustered similarly (Table 3). When considering the entire germplasm as only two populations, the variety between them was 16%. However, when considered as two populations containing a number of sub-populations, 11% of the total variation could be attributed to the variance between the regions, while only 4% was attributed to the variation among populations (Table 3). Overall AMOVA showed that the SNP markers were able to assess the genetic variation among Afghan wheat landraces successfully, revealing clear relationships with regard to their genetic origins.

**Table 3:** **Analysis of molecular variance (AMOVA) for the landraces collected from nine provinces, Badakhshan, Takhar, Ghor, Herat, Kabul, Badghis, Kandahar, Bamyan, and Samangan (1, 2 and 3), according to their geographical distribution, and for all collected landraces with (4) and without (5) controls, according to their year of collection**

**Groups**	**Source of variation**	**Percentage variation** *
1- Nine populations one region	Among populations	14%
	Within populations	86%
2- Two populations one region**	Between populations	16%
	Within populations	84%
3- Nine populations two regions**	Between regions	13%
	Among populations	4%
	Within populations	83%
4- Two populations landraces & controls	Between populations	5%
	Within populations	95%
5- Three populations year of collection	Among populations	12%
	Within populations	88%

*P <0.001.

**Badakhshan and Takhar provinces were considered as one population in (2) and as a single region in (3), while the remaining provinces were considered as a single population in (2) and as a single region in (3).

## Conclusions

This is the first study demonstrating the untapped genetic potential of Dr. Kihara’s Afghan wheat landrace collection. This study was performed using the maximum number of markers available, revealing a substantial degree of genetic diversity among samples. This work also disproved a previous study that evaluated this germplasm and suggested a low degree of overall diversity [26]. Our use of population structure and diversity analyses in combination with collection site data clearly separated the germplasm into distinct groups, which were broadly related to the various agro-ecological zones associated with each geographical region. Use of this phenotypic and environmental data may provide insight into some of the important genetic differences evident among subtypes, indicating possible functional implications for many genetic variations. Preliminary studies have confirmed this hypothesis, identifying potential genotypes associated with biotic and abiotic stress situations. Taken together, the genetic and phenotypic data presented here may help to improve the existing wheat gene pool in Afghanistan, allowing for greater adaptability to local environments, leading to better and more consistent crop yields.

## Methods

### Plant materials

The main source of material used in this study was from the KAWLR collection, which represents a series of Afghan wheat landraces collected from 1950–70 via a variety of scientific expeditions. This collection has been preserved and maintained under the auspices of the National Bio-Resources Project, Japan. A portion of this collection representing 446 unique accessions was selected for use in this study (Additional file 3). Moreover, one durum wheat genotype was also included in theexperiment to ensure that all of the collected materials were hexaploid wheat. In addition to the Afghan varieties, landraces from Iran and Pakistan were also included for use in comparative studies (Additional file 3: Table S2). Healthy seeds were sown in trays and transplanted before winter (November). One-month-old healthy seedling leaves were harvested for DNA extraction.

### Genotyping

Genotyping was performed using genotype by sequencing (GBS) 1.0 V arrays (Triticarte Pvt. Ltd, Australia). SNP markers with a minor allele frequency >10% and markers with >80% good data were selected for further analysis. As the populations represented by this collection are presumed to be homozygous in nature, they were maintained in a controlled pollination environment. Replicates taken from the same landrace that exhibited similar botanical variety phenotypes were considered the same. Genotyping was performed using only a single plant from each landrace. Sequence alignments and SNP extractions were performed using Triticarte software; only SNPs with a call rate >90% were considered. Chromosomal mapping of SNP markers was performed in another recombinant inbred line population (unpublished data) using a Statistical Machine Leaning methodology (Triticarte) [27].

### Data analyses

Genetic diversity analysis was performed using DARwin software [28] and the Jaccard index. The diversity tree was built using a neighbour-joining (NJ) algorithm [29] that relaxes the assumption of equal mutation rates over space and time and produces an un-rooted tree. The confidence interval of the genetic relationships among the accessions was determined by performing 1,000 bootstraps, with the results expressed as percentages at the main nodes of each branch. AMOVA was used to partition the genetic variation into inter- and intra-gene pool diversities based on Arlequin v3.5 software [30]. This analysis was used to identify and separate the samples into collection site-related groups based on a neighbour-joining dendrogram; finally, the results were compared with the morphological characteristics. The statistical significance between mean genetic distances was assessed using the Student’s *t* test. Principal coordinate analysis (PCA) was conducted on the basis of genetic similarity using the EIGEN procedure in GeneAlEx 6.4 [31] to observe the distribution of wheat populations. PCA reduces the original total variance among individuals and clarifies the relationship between two or more characters into a limited number of uncorrelated new variables [32]. This allows visualization of the differences among individuals and identification of possible groups or clusters [33].

A Bayesian-clustering program utilising a Markov Chain Monte Carlo (MCMC) approach, STRUCTURE version 2.3.4 [23], was used to elucidate the structure of genetic variation and identify the number of genetically distinct clusters or gene pools. STRUCTURE was run five independent times for each value of K ranging from 1 to 16 using a burn-in period of 10,000 steps and 100,000 MCMC steps, using a model allowing for admixture and correlated allele frequencies. Parameters were set to their default values, as recommended by the manufacturer [34]. The probability of best fit into each number of assumed clusters (K) was estimated by an ad hoc statistic DK based on the rate of change in the log probability of data between consecutive K values [35]. STRUCTURE analysis was performed again for only 385 genotypes representing nine provinces, each of which contained a minimum of 10 landraces.

## Additional files

Additional file 1:
**Passport details of Afghan wheat landraces preserved in Japan.** The site of collection, respective agro-climatic zones (FAO), latitude, longitude, and collection year for each landrace are shown. Landraces with no clear details regarding the site of collection were reported as unknown. NBRP; National Bio-Resource Project, Japan. Additional file 2:
**Dendrograms for each clade of the landrace germplasm.**
Additional file 3:
**The control varieties used in this study.** NBRP; National Bio-Resource Project, Japan.

## Abbreviations

AMOVA: Analysis of molecular variance
KAWLR: Kihara Afghan wheat landrace
MAF: Minor allele frequency
SNP: Single nucleotide polymorphism

## References

[1] FAO Report. **The world cereal production.** 2011, http://faostat3.fao.org/faostat-gateway/go/to/home/E.

[2] Zeven AC (1998). Landraces: A review of definitions and classification. Euphytica.

[3] Vavilov NI (1926). Centers of origin of cultivated plants. Bull Appl Bot Plant Breed.

[4] Manickavelu A, Niwa S, Ayumi K, Komatsu K, Naruoka Y, Ban T: **Molecular evaluation of Afghan Wheat Landraces.** *Plant Genetic Resources: Characterization and Utilization*, in press.

[5] Tuberosa R, Graner A, Varshney RK (2011). Genomics of plant genetic resources: an introduction. Plant Genetic Resources.

[6] Glaszmann JC, Kilian B, Upadhyaya HD, Varshney RK (2010). Accessing genetic diversity for crop improvement. Curr Opin Plant Biol.

[7] Rafalski JA (2002). Novel genetic mapping tools in plants: SNPs and LD-based approaches. Plant Sci.

[8] Myles S, Peiffer J, Brown PK, Ersoz EE, Zhang Z, Costich DE, Buckler ES (2009). Association mapping: critical considerations shift from genotyping to experimental design. Plant Cell.

[9] Meuwissen THE, Hayes BJ, Goddard ME (2001). Prediction of total genetic value using genome-wide dense marker maps. Genetics.

[10] Yan J, Yang X, Shah T, Sanchez-Villeda H, Li J, Warburton M, Zhou Y, Crouch JH, Xu Y (2010). Highthroughput SNP genotyping with the GoldenGate assay in maize. Mol Breeding.

[11] Sato K, Takeda K (2009). An application of high-throughput SNP genotyping for barley genome mapping and characterization of recombinant chromosome substitution lines. Theor Appl Genet.

[12] Akhunov E, Nicolet C, Dvorak J (2009). Single nucleotide polymorphism genotyping in polyploid wheat with the Illumina GoldenGate assay. Theor Appl Genet.

[13] Wang S, Wong D, Forrest K, Allen A, Chao S, Huang BE, Maccaferri M, Salvi S, Milner SG, Cattivelli L, Mastrangelo AM, Whan A, Stephen S, Barker G, Wieseke R, Plieske J, International Wheat Genome Sequencing Consortium, Lillemo M, Mather D, Appels R, Dolferus R, Brown-Guedira G, Korol A, Akhunova AR, Feuillet C, Salse J, Morgante M, Pozniak C, Luo M, Dvorak J, *et al*.: **Characterization of polyploid wheat genomic diversity using a high-density 90,000 single nucleotide polymorphism array**. *Plant Biotechnol J,* 2014. doi:10.1111/pbi.12183. 10.1111/pbi.12183 PMC4265271 24646323

[14] Mcnally KL, Childs KL, Bohnnert R, Davidson RM, Zhao K, Ulat VJ, Zeller G, Clark RM, Hoen DR, Bureau TE, Stokowski R, Ballinger DG, Frazer KA, Cox DR, Padhukasahasram B, Bustamante CD, Weigel D, Mackill DJ, Bruskiewich RM, Rätsch G, Buell CR, Leung H, Leach JE (2009). Genomewide SNP variation reveals relationships among landraces and modern varieties of rice. Proc Natl Acad Sci U S A.

[15] Cavanagh CR, Chao S, Wang S, Huang BE, Stephen S, Kiani S, Forrest K, Saintenac C, Brown-Guedira GL, Akhunova A, See D, Bai G, Pumphrey M, Tomar L, Wong D, Kong S, Reynolds M, Silva ML, Bockelman H, Talbert L, Anderson JA, Dreisigacker S, Baenziger S, Carter A, Korzun V, Morrell PL, Dubcovsky J, Morell MK, Sorrells ME, Hayden MJ (2013). Genome-wide comparative diversity uncovers multiple targets of selection for improvement in hexaploid wheat landraces and cultivars. Proc Natl Acad Sci U S A.

[16] Mitrofanova OP, Strelchenko PP, Zuev EV, Street K, Konopka J, Mackay M (2013). Genetic diversity of bread wheat landraces collected by scientific expeditions in Afghanistan. Russian J of Genetics: Applied Research.

[17] Buerkert A, Oryakhail M, Filatenko AA, Hammer K (2006). Cultivation and taxonomic classification of wheat landraces in the upper Panjsher valley of Afghanistan after 23 years of war. Genetic Resources Crop Evolution.

[18] Hao C, Wang L, Ge H, Dong Y, Zhand X (2011). Genetic diversity and linkage disequilibrium in Chinese bread wheat (*Triticum aestivum* L.) revealed by SSR markers. PLoS One.

[19] Yamashita K (1965). Cultivated plants and their relatives. Results of the Kyoto University Scientific Expedition to the Karakoram and Hindukush, 1955, Vol. I.

[20] Würschum T, Langer SM, Longin CFH, Korzun V, Akhunov E, Ebmeyer E, Schachschneider R, Schacht J, Kazman E, Reif JC (2013). Population structure, genetic diversity and linkage disequilibrium in elite winter wheat assessed with SNP and SSR markers. Theor Appl Genet.

[21] Sohail Q, Shehzad T, Kilian A, Eltayeb AE, Tanaka H, Tsujimoto H (2012). Development of diversity array technology (DArT) markers for assessment of population structure and diversity in *Aegilops tauschii*. Breeding Sci.

[22] Semagn K, Magorokosho C, Vivek BS, Makumbi D, Beyene Y, Mugo S, Prasanna BM, Warburton ML (2012). Molecular characterization of diverse CIMMYT maize inbred lines from eastern and southern Africa using single nucleotide polymorphic markers. BMC Genomics.

[23] Pritchard JK, Stephens M, Donnelly P (2000). Inference of population structure using multilocus genotype data. Genetics.

[24] Rajaram S, van Ginkel M, Fischer RA: **CIMMYT’s wheat breeding mega-environments (ME).** In *Proceedings of the 8th International wheat genetic symposium, July 19–24, 1993.* Beijing, China.

[25] Reynolds M, Foulkes J, Furbank R, Griffiths S, King J, Murchie E, Parry MJ, Slafer GA (2012). Achieving yield gains in wheat. Plant Cell Environ.

[26] Terasawa Y, Kawahara T, Sasakuma T, Sasanuma T (2009). Evaluation of the genetic diversity of an Afghan wheat collection based on morphological variation, HMW glutenin subunit polymorphisms, and AFLP. Breeding Sci.

[27] Bedo J, Wenzl P, Kowalczyk A, Kilian A (2008). Precision-mapping and statistical validation of quantitative trait loci by machine learning. BMC Genet.

[28] Perrier X, Jacquemoud-Collet JP: **DARwin software.** 2006, http://darwin.cirad.fr/.

[29] Saitou N, Nei M (1987). The neighbor-joining method: a new method for reconstructing phylogenetic trees. Mol Biol Evol.

[30] Excoffier L, Lischer HEL (2010). ARLEQUIN suite ver 3.5: a new series of programs to perform population genetics analyses under Linux and Windows. Mol Ecol Resour.

[31] Peakall R, Smouse PE (2006). GENALEX 6: genetic analysis in Excel. Population genetic software for teaching and research. Molec Ecol Notes.

[32] Wiley RH, Bateson PPG, Klopfer PH (1981). Social structure and individual ontogenies: problems of description, mechanism, and evolution. Perspectives in ethology Volume 4.

[33] Mohammadi SA, Prasanna BM (2003). Analysis of genetic diversity in crop plants - salient statistical tools and considerations. Crop Sci.

[34] Pritchard J, Wen W (2004). Documentation for structure software: version 2.

[35] Evanno G, REGNAUT S, Goudet J (2005). Detecting the number of clusters of individuals using the software structure: a simulation study. Mol Ecol.

