# Supplementary material for: Molecular evaluation of orphan Afghan common wheat (Triticum aestivum L.) landraces collected by Dr. Kihara using single nucleotide polymorphic markers
Source: BMC Plant Biol. 2014 Nov 29;14:320. doi: 10.1186/s12870-014-0320-5 (PMC4255927; doi:10.1186/s12870-014-0320-5)
Supplement: Additional file 1: — Passport details of Afghan wheat landraces preserved in Japan. The site of collection, respective agro-climatic zones (FAO), latitude, longitude, and collection year for each landrace are shown. Landraces with no clear details regarding the site of collection were reported as unknown. NBRP; National Bio-Resource Project, Japan. [file 12870_2014_320_MOESM1_ESM.pdf]

Supplementary Table 1. Passport details of Afghan wheat landraces preserved in Japan

Entry no.	Gene bank acc. no	Collection site	Province	Agro-climatic zone	Longitude	Latitude	Altitude by NBRP (MSL)	Storage year
501	KU11201	Bazar of Kabul	Kabul	F	69.171703	34.528455	-	1979
502	KU11202A	Bazar of Kabul	Kabul	F	69.171703	34.528455	-	1979
503	KU11202Ba	Bazar of Kabul	Kabul	F	69.171703	34.528455	-	1979
504	KU11202Bb	Bazar of Kabul	Kabul	F	69.171703	34.528455	-	1979
505	KU11203	8 km N of Kunduz to Eman Sahab	Kunduz	B	68.80188	36.79609	-	1979
506	KU11204	8 km N of Kunduz to Eman Sahab	Kunduz	B	68.80188	36.79609	-	1979
507	KU11205	8 km N of Kunduz to Eman Sahab	Kunduz	B	68.80188	36.79609	-	1979
508	KU11206	Chinzaii E of Khanabad Takhar	Takhar	B	69.112244	36.702559	640	1979
509	KU11207	Chinzaii E of Khanabad Takhar	Takhar	B	69.112244	36.702559	640	1979
510	KU11208A	Chinzaii E of Khanabad Takhar	Takhar	B	69.112244	36.702559	640	1979
511	KU11208B	Chinzaii E of Khanabad Takhar	Takhar	B	69.112244	36.702559	640	1979
512	KU11209	Chinzaii E of Khanabad Takhar	Takhar	B	69.112244	36.702559	640	1979
513	KU11210	Chinzaii E of Khanabad Takhar	Takhar	B	69.112244	36.702559	640	1979
514	KU11211	13.2 km S of Farha Takhar	Takhar	B	62.1123	32.3754	1300	1979
515	KU11212	13.2 km S of Farha Takhar	Takhar	B	62.1123	32.3754	1300	1979
517	KU11214	37 km E of Taluquan near Kalafgan Takhar	Takhar	B	69.9026	36.7631	1370	1979
518	KU11215	37 km E of Taluquan near Kalafgan Takhar	Takhar	B	69.9026	36.7631	1370	1979
519	KU11216A	37 km E of Taluquan near Kalafgan Takhar	Takhar	B	69.9026	36.7631	1370	1979
520	KU11216B	37 km E of Taluquan near Kalafgan Takhar	Takhar	B	69.9026	36.7631	1370	1979
521	KU11217	41 km E of Taluquan near Karafgan Takhar	Takhar	B	69.529724	36.79609	1530	1979
522	KU11218	47 km E of Taluquan near Karafgan Takhar	Takhar	B	69.529724	36.79609	1580	1979
523	KU11219	47 km E of Taluquan near Karafgan Takhar	Takhar	B	69.529724	36.79609	1580	1979
524	KU11221A	6 km W of Kisim Badakhshan	Badakhshan	B	70.811995	36.734772	1060	1979
525	KU11221B	6 km W of Kisim Badakhshan	Badakhshan	B	70.811995	36.734772	1060	1979
526	KU11222	6 km W of Kisim Badakhshan	Badakhshan	B	70.811995	36.734772	1060	1979
527	KU11223	6 km W of Kisim Badakhshan	Badakhshan	B	70.811995	36.734772	1060	1979
528	KU11224A	6 km W of Kisim Badakhshan	Badakhshan	B	70.811995	36.734772	1060	1979
529	KU11224B	6 km W of Kisim Badakhshan	Badakhshan	B	70.811995	36.734772	1060	1979
530	KU11225	8 km S of Faizabad Badakhshan	Badakhshan	B	70.5132	37.147	1230	1979
531	KU11226	8 km S of Faizabad Badakhshan	Badakhshan	B	70.5132	37.147	1230	1979
532	KU11227	8 km S of Faizabad Badakhshan	Badakhshan	B	70.5132	37.147	1230	1979
533	KU11228	16 km SE of Faizabad Badakhshan	Badakhshan	B	70.5132	37.147	1300	1979
534	KU11229	16 km SE of Faizabad Badakhshan	Badakhshan	B	70.5132	37.147	1300	1979

**535**	KU11230A	16 km SE of Faizabad Badakhshan	Badakhshan	B	70.5132	37.147	1300	1979
**536**	KU11230B	16 km SE of Faizabad Badakhshan	Badakhshan	B	70.5132	37.147	1300	1979
**537**	KU11231	Sarai Jamarad 13 km NW of Barak Badakhshan	Badakhshan	B	66.461792	35.101934	1390	1979
**538**	KU11232	Sarai Jamarad 13 km NW of Barak Badakhshan	Badakhshan	B	66.461792	35.101934	1390	1979
**539**	KU11233	8 km NW of Barak Badakhshan	Badakhshan	B	69.6	35.35	1450	1979
**540**	KU11234	8 km NW of Barak Badakhshan	Badakhshan	B	69.6	35.35	1450	1979
**541**	KU11235	8 km NW of Barak Badakhshan	Badakhshan	B	69.6	35.35	1450	1979
**542**	KU11236A	8 km NW of Barak Badakhshan	Badakhshan	B	69.6	35.35	1450	1979
**543**	KU11236B	8 km NW of Barak Badakhshan	Badakhshan	B	69.6	35.35	1450	1979
**544**	KU11237A	Puli-zeribon Shewa Badakhshan	Badakhshan	B	70.488281	33.266824	2540	1979
**545**	KU11237B	Puli-zeribon Shewa Badakhshan	Badakhshan	B	70.488281	33.266824	2540	1979
**546**	KU11238	Puli-zeribon Shewa Badakhshan	Badakhshan	B	70.488281	33.266824	2540	1979
**547**	KU11239Aa	Puli-zeribon Shewa Badakhshan	Badakhshan	B	70.488281	33.266824	2540	1979
**548**	KU11239Ab	Puli-zeribon Shewa Badakhshan	Badakhshan	B	70.488281	33.266824	2540	1979
**549**	KU11240A	Bamdara 13 km N of Robobi to Shewa Badakhshan	Badakhshan	B	68.9377778	35.9277778	1680	1979
**550**	KU11240B	Bamdara 13 km N of Robobi to Shewa Badakhshan	Badakhshan	B	68.9377778	35.9277778	1680	1979
**551**	KU11241A	Bamdara 13 km N of Robobi to Shewa Badakhshan	Badakhshan	B	68.9377778	35.9277778	1680	1979
**552**	KU11241B	Bamdara 13 km N of Robobi to Shewa Badakhshan	Badakhshan	B	68.9377778	35.9277778	1680	1979
**553**	KU11242A	Bamdara 13 km N of Robobi to Shewa Badakhshan	Badakhshan	B	68.9377778	35.9277778	1680	1979
**554**	KU11242B	Bamdara 13 km N of Robobi to Shewa Badakhshan	Badakhshan	B	68.9377778	35.9277778	1680	1979
**555**	KU11243A	Bamdara 13 km N of Robobi to Shewa Badakhshan	Badakhshan	B	68.9377778	35.9277778	1680	1979
**556**	KU11243B	Bamdara 13 km N of Robobi to Shewa Badakhshan	Badakhshan	B	68.9377778	35.9277778	1680	1979
**557**	KU11244	Bamdara 13 km N of Robobi to Shewa Badakhshan	Badakhshan	B	68.9377778	35.9277778	1680	1979
**558**	KU11245	Yardar 4 km SE of Barak Badakhshan	Badakhshan	B	70.883789	37.753344	1430	1979
**559**	KU11246	Yardar ca. 5 km SE of Barak Badakhshan	Badakhshan	B	70.883789	37.753344	1460	1979
**560**	KU11247	Yardar ca. 5 km SE of Barak Badakhshan	Badakhshan	B	70.883789	37.753344	1460	1979
**561**	KU11248	Yardar ca. 5 km SE of Barak Badakhshan	Badakhshan	B	70.883789	37.753344	1460	1979
**562**	KU11249A	Yardar ca. 5 km SE of Barak Badakhshan	Badakhshan	B	70.883789	37.753344	1460	1979
**563**	KU11249B	Yardar ca. 5 km SE of Barak Badakhshan	Badakhshan	B	70.883789	37.753344	1460	1979
**564**	KU11250A	6 km S of Barak Badakhshan	Badakhshan	B	69.6	35.35	1460	1979
**565**	KU11250B	6 km S of Barak Badakhshan	Badakhshan	B	69.6	35.35	1460	1979
**566**	KU11251	6 km S of Barak Badakhshan	Badakhshan	B	69.6	35.35	1460	1979
**567**	KU11252A	6 km S of Barak Badakhshan	Badakhshan	B	69.6	35.35	1460	1979
**568**	KU11252B	6 km S of Barak Badakhshan	Badakhshan	B	69.6	35.35	1460	1979
**569**	KU11253	8 km S of Barak Badakhshan	Badakhshan	B	69.6	35.35	1450	1979
**570**	KU11254A	8 km S of Barak Badakhshan	Badakhshan	B	69.6	35.35	1450	1979
**571**	KU11254B	8 km S of Barak Badakhshan	Badakhshan	B	69.6	35.35	1450	1979

**572**	KU11255A	13 km S of Barak Badakhshan	Badakhshan	B	69.6	35.35	1460	1979
**573**	KU11255B	13 km S of Barak Badakhshan	Badakhshan	B	69.6	35.35	1460	1979
**574**	KU11256	13 km S of Barak Badakhshan	Badakhshan	B	69.6	35.35	1460	1979
**575**	KU11257A	13 km S of Barak Badakhshan	Badakhshan	B	69.6	35.35	1460	1979
**577**	KU11258	13 km S of Barak Badakhshan	Badakhshan	B	69.6	35.35	1460	1979
**578**	KU11259	Chinge Peyong 2.5 km N of Jurum Badakhshan	Badakhshan	B	70.9613889	34.1077778	1440	1979
**579**	KU11260	Chinge Peyong 2.5 km N of Jurum Badakhshan	Badakhshan	B	70.9613889	34.1077778	1440	1979
**580**	KU11261	Chinge Peyong 2.5 km N of Jurum Badakhshan	Badakhshan	B	70.9613889	34.1077778	1440	1979
**581**	KU11262	Khanadab 2.5 km S of Jurum Badakhshan	Badakhshan	B	69.112244	36.702559	-	1979
**582**	KU11263	Khanadab 2.5 km S of Jurum Badakhshan	Badakhshan	B	69.112244	36.702559	-	1979
**583**	KU11264	Khanadab 2.5 km S of Jurum Badakhshan	Badakhshan	B	69.112244	36.702559	-	1979
**584**	KU11265A	Khanadab 2.5 km S of Jurum Badakhshan	Badakhshan	B	69.112244	36.702559	-	1979
**585**	KU11265B	Khanadab 2.5 km S of Jurum Badakhshan	Badakhshan	B	69.112244	36.702559	-	1979
**586**	KU11266	Khanadab 2.5 km S of Jurum Badakhshan	Badakhshan	B	69.112244	36.702559	-	1979
**587**	KU11267	5 km S of Jurum Badakhshan	Badakhshan	B	70.82	36.849998	1490	1979
**588**	KU11268	5 km S of Jurum Badakhshan	Badakhshan	B	70.82	36.849998	1490	1979
**589**	KU11269	5 km S of Jurum Badakhshan	Badakhshan	B	70.82	36.849998	1490	1979
**590**	KU11270	5 km S of Jurum Badakhshan	Badakhshan	B	70.82	36.849998	1490	1979
**591**	KU11271A	5 km S of Jurum Badakhshan	Badakhshan	B	70.82	36.849998	1490	1979
**592**	KU11271B	5 km S of Jurum Badakhshan	Badakhshan	B	70.82	36.849998	1490	1979
**593**	KU11272	North of Jurum Badakhshan	Badakhshan	B	70.82	36.849998	1570	1979
**594**	KU11273	North of Jurum Badakhshan	Badakhshan	B	70.82	36.849998	1570	1979
**595**	KU11274	North of Jurum Badakhshan	Badakhshan	B	70.82	36.849998	1570	1979
**596**	KU11275	North of Jurum Badakhshan	Badakhshan	B	70.82	36.849998	1570	1979
**597**	KU11276	North of Jurum Badakhshan	Badakhshan	B	70.82	36.849998	1570	1979
**598**	KU11277	North of Jurum Badakhshan	Badakhshan	B	70.82	36.849998	1570	1979
**599**	KU11278A	1.5 km E of Barak Badakhshan	Badakhshan	B	69.6	35.35	1500	1979
**600**	KU11278B	1.5 km E of Barak Badakhshan	Badakhshan	B	69.6	35.35	1500	1979
**601**	KU11279A	1.5 km E of Barak Badakhshan	Badakhshan	B	69.6	35.35	1500	1979
**602**	KU11279B	1.5 km E of Barak Badakhshan	Badakhshan	B	69.6	35.35	1500	1979
**605**	KU11282A	1.5 km E of Barak Badakhshan	Badakhshan	B	69.6	35.35	1500	1979
**606**	KU11282B	1.5 km E of Barak Badakhshan	Badakhshan	B	69.6	35.35	1500	1979
**607**	KU11283A	1.5 km E of Barak Badakhshan	Badakhshan	B	69.6	35.35	1500	1979
**608**	KU11283B	1.5 km E of Barak Badakhshan	Badakhshan	B	69.6	35.35	1500	1979
**609**	KU11284	1.5 km E of Barak Badakhshan	Badakhshan	B	69.6	35.35	1500	1979
**611**	KU11286	2.5 km E of Barak Badakhshan	Badakhshan	B	69.6	35.35	1540	1979
**612**	KU11287	2.5 km E of Barak Badakhshan	Badakhshan	B	69.6	35.35	1540	1979

613	KU11288	2.5 km E of Barak Badakhshan	Badakhshan	B	69.6	35.35	1540	1979
614	KU11289	4 km E of Barak Badakhshan	Badakhshan	B	69.6	35.35	1530	1979
615	KU11290	Pijoj 13 km E of Barak Badakhshan	Badakhshan	B	66.461792	35.101934	1750	1979
616	KU11291	Pijoj 13 km E of Barak Badakhshan	Badakhshan	B	66.461792	35.101934	1750	1979
617	KU11292	Pijoj 13 km E of Barak Badakhshan	Badakhshan	B	66.461792	35.101934	1750	1979
618	KU11293	20 km E of Barak Badakhshan	Badakhshan	B	69.6	35.35	1800	1979
619	KU11294	Rezwon 27 km E of Barak Badakhshan	Badakhshan	B	66.461792	35.101934	1940	1979
620	KU11295A	Rezwon 27 km E of Barak Badakhshan	Badakhshan	B	66.461792	35.101934	1940	1979
621	KU11295B	Rezwon 27 km E of Barak Badakhshan	Badakhshan	B	66.461792	35.101934	1940	1979
622	KU11296	Rezwon 27 km E of Barak Badakhshan	Badakhshan	B	66.461792	35.101934	1940	1979
623	KU11297A	Rezwon 27 km E of Barak Badakhshan	Badakhshan	B	66.461792	35.101934	1940	1979
624	KU11297B	Rezwon 27 km E of Barak Badakhshan	Badakhshan	B	66.461792	35.101934	1940	1979
625	KU11298A	Rezwon 27 km E of Barak Badakhshan	Badakhshan	B	66.461792	35.101934	1940	1979
626	KU11298Ba	Rezwon 27 km E of Barak Badakhshan	Badakhshan	B	66.461792	35.101934	1940	1979
627	KU11298Bb	Rezwon 27 km E of Barak Badakhshan	Badakhshan	B	66.461792	35.101934	1940	1979
628	KU11299	Rezwon 27 km E of Barak Badakhshan	Badakhshan	B	66.461792	35.101934	1940	1979
629	KU11300A	Rezwon 27 km E of Barak Badakhshan	Badakhshan	B	66.461792	35.101934	1940	1979
630	KU11300B	Rezwon 27 km E of Barak Badakhshan	Badakhshan	B	66.461792	35.101934	1940	1979
631	KU11301A	Rezwon 27 km E of Barak Badakhshan	Badakhshan	B	66.461792	35.101934	1940	1979
632	KU11301B	Rezwon 27 km E of Barak Badakhshan	Badakhshan	B	66.461792	35.101934	1940	1979
633	KU11302	29 km E of Barak Badakhshan	Badakhshan	B	69.6	35.35	1960	1979
634	KU11303	29 km E of Barak Badakhshan	Badakhshan	B	69.6	35.35	1960	1979
635	KU11304	29 km E of Barak Badakhshan	Badakhshan	B	69.6	35.35	1960	1979
636	KU11305	29 km E of Barak Badakhshan	Badakhshan	B	69.6	35.35	1960	1979
637	KU11306	37 km E of Barak Badakhshan	Badakhshan	B	69.6	35.35	2100	1979
638	KU11307	37 km E of Barak Badakhshan	Badakhshan	B	69.6	35.35	2100	1979
640	KU11309	37 km E of Barak Badakhshan	Badakhshan	B	69.6	35.35	2100	1979
641	KU11310	Pasibo 13 km SE of Barak Badakhshan	Badakhshan	B	66.461792	35.101934	1540	1979
642	KU11311	Pasibo 13 km SE of Barak Badakhshan	Badakhshan	B	66.461792	35.101934	1540	1979
643	KU11312A	Zu 30 km SE of Barak Badakhshan	Badakhshan	B	67.785645	34.723555	1750	1979
644	KU11312B	Zu 30 km SE of Barak Badakhshan	Badakhshan	B	67.785645	34.723555	1750	1979
645	KU11313	Zu 30 km SE of Barak Badakhshan	Badakhshan	B	67.785645	34.723555	1750	1979
646	KU11314A	Zu 30 km SE of Barak Badakhshan	Badakhshan	B	67.785645	34.723555	1750	1979
647	KU11314B	Zu 30 km SE of Barak Badakhshan	Badakhshan	B	67.785645	34.723555	1750	1979
648	KU11315	Zu 30 km SE of Barak Badakhshan	Badakhshan	B	67.785645	34.723555	1750	1979
649	KU11316	Yomal 34 km SE of Barak Badakhshan	Badakhshan	B	66.461792	35.101934	1810	1979
650	KU11317A	Yomal 34 km SE of Barak Badakhshan	Badakhshan	B	66.461792	35.101934	1810	1979

**651**	KU11317B	Yomal 34 km SE of Barak Badakhshan	Badakhshan	B	66.461792	35.101934	1810	1979
**652**	KU11318	Tirgaran 46 km SE of Barak Badakhshan	Badakhshan	B	70.9833333	36.1791666	2040	1979
**653**	KU11319A	Tirgaran 46 km SE of Barak Badakhshan	Badakhshan	B	70.9833333	36.1791666	2040	1979
**654**	KU11319B	Tirgaran 46 km SE of Barak Badakhshan	Badakhshan	B	70.9833333	36.1791666	2040	1979
**655**	KU11320	Tirgaran 46 km SE of Barak Badakhshan	Badakhshan	B	70.9833333	36.1791666	2040	1979
**657**	KU11322	Tirgaran ca. 49 km SE of Barak to Zebak Badakhshan	Badakhshan	B	70.9833333	36.1791666	2120	1979
**658**	KU11323	Tirgaran ca. 49 km SE of Barak to Zebak Badakhshan	Badakhshan	B	70.9833333	36.1791666	2120	1979
**659**	KU11324A	Tirgaran ca. 49 km SE of Barak to Zebak Badakhshan	Badakhshan	B	70.9833333	36.1791666	2120	1979
**660**	KU11324B	Tirgaran ca. 49 km SE of Barak to Zebak Badakhshan	Badakhshan	B	70.9833333	36.1791666	2120	1979
**661**	KU11325	Shakelan 33 km SE of Barak to Zebak Badakhshan	Badakhshan	B	71.38916	36.93233	1650	1979
**662**	KU11326	Shakelan 33 km SE of Barak to Zebak Badakhshan	Badakhshan	B	71.38916	36.93233	1650	1979
**663**	KU11327	Shakelan 33 km SE of Barak to Zebak Badakhshan	Badakhshan	B	71.38916	36.93233	1650	1979
**664**	KU11328	Shakelan 33 km SE of Barak to Zebak Badakhshan	Badakhshan	B	71.38916	36.93233	1650	1979
**665**	KU11329	Shakelan 33 km SE of Barak to Zebak Badakhshan	Badakhshan	B	71.38916	36.93233	1650	1979
**666**	KU11330	Awan 25 km SE of Barak to Zebak Badakhshan	Badakhshan	B	70.905	36.6831	1750	1979
**667**	KU11331A	Awan 25 km SE of Barak to Zebak Badakhshan	Badakhshan	B	70.905	36.6831	1750	1979
**668**	KU11331B	Awan 25 km SE of Barak to Zebak Badakhshan	Badakhshan	B	70.905	36.6831	1750	1979
**669**	KU11332	Awan 25 km SE of Barak to Zebak Badakhshan	Badakhshan	B	70.905	36.6831	1760	1979
**670**	KU11333	Yakinjone Wordu ca. 20 km SE of Barak to Zebak Badakhshan	Badakhshan	B	66.461792	35.101934	1620	1979
**672**	KU11334B	North of Puli-zeribon Shewa Badakhshan	Badakhshan	B	70.488281	33.266824	-	1979
**673**	KU11335A	North of Puli-zeribon Shewa Badakhshan	Badakhshan	B	70.488281	33.266824	-	1979
**674**	KU11335B	North of Puli-zeribon Shewa Badakhshan	Badakhshan	B	70.488281	33.266824	-	1979
**675**	KU11336A	North of Puli-zeribon Shewa Badakhshan	Badakhshan	B	70.488281	33.266824	-	1979
**676**	KU11336B	North of Puli-zeribon Shewa Badakhshan	Badakhshan	B	70.488281	33.266824	-	1979
**677**	KU11337	Chapchi Magzar Barak Badakhshan	Badakhshan	B	68.1261111	34.5766667	1460	1979
**678**	KU11338	Chapchi Magzar Barak Badakhshan	Badakhshan	B	68.1261111	34.5766667	1460	1979
**679**	KU11339	Chapchi Magzar Barak Badakhshan	Badakhshan	B	68.1261111	34.5766667	1460	1979
**680**	KU11340	Chapchi Magzar Barak Badakhshan	Badakhshan	B	68.1261111	34.5766667	1460	1979
**681**	KU11341	Chapchi Magzar Barak Badakhshan	Badakhshan	B	68.1261111	34.5766667	1460	1979
**682**	KU11346	Bandi Amir	Bamyan	E	67.2147222	34.8436111	-	1979
**683**	KU11347	Bandi Amir	Bamyan	E	67.2147222	34.8436111	-	1979
**684**	KU3045	Suburbs of Kandahar	Kandahar	H	65.7615	31.6031	-	1956
**685**	KU3046	Suburbs of Kandahar	Kandahar	H	65.7615	31.6031	-	1956
**686**	KU3047	Kandahar - Jaldak	Kandahar	H	65.7615	31.6031	-	1956
**687**	KU3049	Kandahar - Jaldak	Kandahar	H	65.7615	31.6031	-	1956
**688**	KU3050	Kandahar - Jaldak	Kandahar	H	65.7615	31.6031	-	1956
**689**	KU3051	Kandahar - Jaldak	Kandahar	H	65.7615	31.6031	-	1956

**690**	KU3053	Kandahar - Jaldak	Kandahar	H	65.7615	31.6031	-	1956
**691**	KU3054	Jaldak	Zabul	H	66.7333	31.9735	-	1956
**692**	KU3056	Jaldak - Ghazni	Ghazni	D	66.7333	31.9735	-	1956
**693**	KU3057	Jaldak - Ghazni	Ghazni	D	66.7333	31.9735	-	1956
**694**	KU3059	Jaldak - Ghazni	Ghazni	D	66.7333	31.9735	-	1956
**695**	KU3060	Jaldak - Ghazni	Ghazni	D	66.7333	31.9735	-	1956
**696**	KU3062	Kabul	Kabul	F	69.168835	34.528141	-	1956
**697**	KU3064	Kabul	Kabul	F	69.168835	34.528141	-	1956
**698**	KU3066	Kabul	Kabul	F	69.168835	34.528141	-	1956
**699**	KU3067	Kabul	Kabul	F	69.168835	34.528141	-	1956
**700**	KU3068	Kabul	Kabul	F	69.168835	34.528141	-	1956
**701**	KU3069	Kabul	Kabul	F	69.168835	34.528141	-	1956
**702**	KU3070	Kabul	Kabul	F	69.168835	34.528141	-	1956
**703**	KU3071	Kabul	Kabul	F	69.168835	34.528141	-	1956
**704**	KU3074	Kabul	Kabul	F	69.168835	34.528141	-	1956
**705**	KU3076	Kabul	Kabul	F	69.168835	34.528141	-	1956
**706**	KU3077	Kabul	Kabul	F	69.168835	34.528141	-	1956
**707**	KU3078	unknown	unknown	unknown	69.171181	34.532751	-	1956
**708**	KU3079	unknown	unknown	unknown	69.171181	34.532751	-	1956
**710**	KU3081	Kabul - Doshi	Kabul	F	69.168835	34.528141	-	1956
**711**	KU3082	Kabul - Doshi	Kabul	F	69.168835	34.528141	-	1956
**712**	KU3083	17 km N of Doshi (Doshi - Pul-i-Khumri)	Baghlan	B	68.732758	35.640111	-	1956
**713**	KU3084	12 km S of Pul-i-Khumri(Doshi - Pul-i-Khumri)	Baghlan	B	68.722115	35.87197	-	1956
**714**	KU3085	Andkhui - Maimana	Faryabu	A	65.120373	36.925551	-	1956
**715**	KU3086	Andkhui - Maimana	Faryabu	A	65.120373	36.925551	-	1956
**716**	KU3087	Andkhui - Maimana	Faryabu	A	65.120373	36.925551	-	1956
**717**	KU3088	Andkhui - Maimana	Faryabu	A	65.120373	36.925551	-	1956
**718**	KU3089	Maimana	Faryabu	A	64.761991	35.930657	-	1956
**719**	KU3090	Maimana	Faryabu	A	64.761991	35.930657	-	1956
**720**	KU3091	Maimana	Faryabu	A	64.761991	35.930657	-	1956
**721**	KU3092	Maimana	Faryabu	A	64.761991	35.930657	-	1956
**722**	KU7428	Tunian E of Herat Herat	Herat	C	62.532577	34.310887	1240	1967
**723**	KU7430	Palpini Herat	Herat	C	62.202988	34.369851	1210	1967
**724**	KU7432	1.5 km E of Obeh (1.7 km N of Hari rud River) Herat	Herat	C	65.257873	32.274593	1360	1967
**725**	KU7434A	10 km E of Karokh NE of Herat Herat	Herat	C	62.579269	34.541971	1390	1967
**726**	KU7434B	10 km E of Karokh NE of Herat Herat	Herat	C	62.579269	34.541971	1390	1967
**727**	KU7435	10 km E of Karokh NE of Herat Herat	Herat	C	62.579269	34.541971	1390	1967

728	KU7436	8.3 km W of Armalik Herat	Herat	C	62.190113	34.376681	1460	1967
729	KU7437	1 km E of Armalik Herat	Herat	C	69.171181	34.532751	1490	1967
730	KU7438	1 km E of Armalik Herat	Herat	C	69.171181	34.532751	1490	1967
731	KU7441	4 km E of Armalik Herat	Herat	C	62.190113	34.376681	1550	1967
732	KU7442	18 km E of Armalik Herat	Herat	C	62.190113	34.376681	1670	1967
733	KU7443	18 km E of Armalik Herat	Herat	C	62.190113	34.376681	1670	1967
734	KU7444	18 km E of Armalik Herat	Herat	C	62.190113	34.376681	1670	1967
735	KU7445	18 km E of Armalik Herat	Herat	C	62.190113	34.376681	1670	1967
736	KU7446A	18 km E of Armalik Herat	Herat	C	62.190113	34.376681	1670	1967
737	KU7446B	18 km E of Armalik Herat	Herat	C	62.190113	34.376681	1670	1967
738	KU7447	18 km E of Armalik Herat	Herat	C	62.190113	34.376681	1670	1967
739	KU7448	20 km E of Armalik Herat	Herat	C	62.190113	34.376681	1730	1967
740	KU7449	20 km E of Armalik Herat	Herat	C	62.190113	34.376681	1730	1967
741	KU7450	25 km E of Armalik Herat	Herat	C	62.190113	34.376681	1790	1967
742	KU7451	28 km E of Armalik Herat	Herat	C	62.190113	34.376681	1850	1967
743	KU7452	33 km E of Armalik Herat	Herat	C	62.190113	34.376681	1970	1967
744	KU7453	34 km E of Armalik Herat	Herat	C	62.190113	34.376681	1970	1967
745	KU7455	46 km E of Armalik Herat	Herat	C	62.190113	34.376681	2270	1967
746	KU7456	63 km E of Armalik Herat	Herat	C	62.190113	34.376681	1640	1967
747	KU7457	63 km E of Armalik Herat	Herat	C	62.190113	34.376681	1640	1967
748	KU7458	7 km N of Chekao Badghis	Badghis	C	63.070393	34.67989	1270	1967
749	KU7459	1.5 km W of Qala Nau Badghis	Badghis	C	63.12973	34.990179	910	1967
750	KU7460	1.5 km W of Qala Nau Badghis	Badghis	C	63.12973	34.990179	910	1967
751	KU7461	6.6 km SE of Qala Nau Badghis	Badghis	C	63.12973	34.990179	970	1967
752	KU7462	18 km SE of Qala Nau Badghis	Badghis	C	63.12973	34.990179	1060	1967
753	KU7463	35 km SE of Qala Nau Badghis	Badghis	C	63.12973	34.990179	1180	1967
754	KU7464	35 km SE of Qala Nau Badghis	Badghis	C	63.12973	34.990179	1180	1967
755	KU7465	35 km SE of Qala Nau Badghis	Badghis	C	63.12973	34.990179	1180	1967
756	KU7466	Qaddis SE of Qala Nau Badghis	Badghis	C	63.396206	35.070553	1240	1967
757	KU7467	30 km NW of Qaddis (11 km SE of Qala Nau) Badghis	Badghis	C	63.396206	35.070553	1060	1967
758	KU7468	16 km SE of Yakhak (SE of Qala Nau) Badghis	Badghis	C	63.264313	34.609023	2240	1967
759	KU7469	16 km SE of Yakhak (SE of Qala Nau) Badghis	Badghis	C	63.264313	34.609023	2240	1967
760	KU7470	Qaddis (SE of Qala Nau) Badghis	Badghis	C	63.396206	35.070553	1240	1967
761	KU7471	18 km SE of Yakhak (SE of Qala Nau) Badghis	Badghis	C	63.264313	34.609023	2180	1967
762	KU7472	20 km SE of Yakhak (SE of Qala Nau) Badghis	Badghis	C	63.264313	34.609023	2150	1967
763	KU7473	20 km SE of Yakhak (SE of Qala Nau) Badghis	Badghis	C	63.264313	34.609023	2150	1967
764	KU7475	20 km SE of Yakhak (SE of Qala Nau) Badghis	Badghis	C	63.264313	34.609023	2150	1967

765	KU7476	3 km W of Karakh (NE of Herat) Herat	Herat	C	66.038361	32.331934	1300	1967
766	KU7479	66 km S of Herat Herat	Herat	C	62.082825	34.285815	1490	1967
767	KU7480	Junction of Farah rud and Khorbordar River Ghor	Ghor	C	64.845886	34.113169	910	1967
768	KU7481	Junction of Farah rud and Khorbordar River Ghor	Ghor	C	64.845886	34.113169	910	1967
769	KU7482	Junction of Farah rud and Khorbordar River Ghor	Ghor	C	64.845886	34.113169	910	1967
770	KU7483	108 km S of Herat Herat	Herat	C	62.082825	34.285815	1120	1967
771	KU7484	63 km S of Herat Herat	Herat	C	62.082825	34.285815	1360	1967
772	KU7486	13 km N of Obeh Herat	Herat	C	65.257873	32.274593	1420	1967
773	KU7487	13 km N of Obeh Herat	Herat	C	65.257873	32.274593	1420	1967
774	KU7489	36 km W of Besha Herat	Herat	C	69.153385	34.540868	1360	1967
776	KU7491	26 km W of Besha Herat	Herat	C	69.153385	34.540868	1420	1967
777	KU7493	13 km W of Besha Herat	Herat	C	69.153385	34.540868	1520	1967
778	KU7494	13 km W of Besha Herat	Herat	C	69.153385	34.540868	1520	1967
779	KU7495	13 km W of Besha Herat	Herat	C	69.153385	34.540868	1520	1967
780	KU7496	1.6 km S of Dakhan Herat	Herat	C	63.434601	33.950879	1550	1967
781	KU7497	1.6 km S of Dakhan Herat	Herat	C	63.434601	33.950879	1550	1967
782	KU7498	1.6 km S of Dakhan Herat	Herat	C	63.434601	33.950879	1550	1967
783	KU7499	1.6 km S of Dakhan Herat	Herat	C	63.434601	33.950879	1550	1967
784	KU7502	3 km SE of Margha Ghor	Ghor	C	64.254885	34.347872	1880	1967
785	KU7503	3 km SE of Margha Ghor	Ghor	C	64.254885	34.347872	1880	1967
786	KU7504	3 km SE of Margha Ghor	Ghor	C	64.254885	34.347872	1880	1967
787	KU7505	5 km SE of Margha Ghor	Ghor	C	64.254885	34.347872	2120	1967
788	KU7506	16 km SE of Margha Ghor	Ghor	C	64.254885	34.347872	2240	1967
790	KU7508	16 km SE of Margha Ghor	Ghor	C	64.254885	34.347872	2240	1967
791	KU7509	16 km SE of Margha Ghor	Ghor	C	64.254885	34.347872	2240	1967
792	KU7510	16 km SE of Margha Ghor	Ghor	C	64.254885	34.347872	2240	1967
793	KU7511	16 km SE of Margha Ghor	Ghor	C	64.254885	34.347872	2240	1967
794	KU7512	10 km NW of Targi-Azao Ghor	Ghor	C	64.914379	34.100235	2490	1967
795	KU7513	10 km NW of Targi-Azao Ghor	Ghor	C	64.914379	34.100235	2490	1967
796	KU7514	10 km NW of Targi-Azao Ghor	Ghor	C	64.914379	34.100235	2490	1967
797	KU7515	15 km NW of Targi-Azao Ghor	Ghor	C	64.914379	34.100235	2490	1967
798	KU7516	Tagao Ishan River valley 3 km E of Qala Hissar W of Qala Shararak Ghor	Ghor	C	64.048805	34.163125	2180	1967
799	KU7517	Tagao Ishan River valley 3 km E of Qala Hissar W of Qala Shararak Ghor	Ghor	C	64.048805	34.163125	2180	1967
800	KU7518	Tagao Ishan River valley 3 km E of Qala Hissar W of Qala Shararak Ghor	Ghor	C	64.048805	34.163125	2180	1967
802	KU7520	Tagao Ishan River valley 3 km E of Qala Hissar W of Qala Shararak Ghor	Ghor	C	64.048805	34.163125	2180	1967
803	KU7521	Tagao Ishan River valley 3 km E of Qala Hissar W of Qala Shararak Ghor	Ghor	C	64.048805	34.163125	2180	1967
804	KU7522	3 km S of Tagao Ishan valley E of Qala Hissar W of Qala Shararak Ghor	Ghor	C	64.048805	34.163125	2300	1967

**805**	KU7523	3 km E of Qala Hissar (W of Qala Shararak) on Tagao Ishan River Ghor	Ghor	C	64.048805	34.163125	2180	1967
**806**	KU7524	3 km E of Qala Hissar (W of Qala Shararak) on Tagao Ishan River Ghor	Ghor	C	64.048805	34.163125	2180	1967
**807**	KU7525	3 km S of Tangi Azao near Qala Shararak Ghor	Ghor	C	64.225702	34.119948	2390	1967
**808**	KU7526	3 km S of Tangi Azao near Qala Shararak Ghor	Ghor	C	64.225702	34.119948	2390	1967
**809**	KU7527	11 km W of Qala Shararak Ghor	Ghor	C	64.225702	34.119948	2270	1967
**810**	KU7528	83 km SW of Djam near Qala Shararak Ghor	Ghor	C	64.225702	34.119948	2390	1967
**811**	KU7529	83 km SW of Djam near Qala Shararak Ghor	Ghor	C	64.225702	34.119948	2390	1967
**812**	KU7530	83 km SW of Djam near Qala Shararak Ghor	Ghor	C	64.225702	34.119948	2390	1967
**813**	KU7531	3 km S of Gok Tago near Shararak Ghor	Ghor	C	64.239607	34.122648	2300	1967
**814**	KU7532	15 km NE of Qala Shararak Ghor	Ghor	C	64.225702	34.119948	2610	1967
**815**	KU7533	6 km E of Qala Shararak Ghor	Ghor	C	64.225702	34.119948	2390	1967
**816**	KU7534	6 km E of Qala Shararak Ghor	Ghor	C	64.225702	34.119948	2390	1967
**817**	KU7535	6 km E of Qala Shararak Ghor	Ghor	C	64.225702	34.119948	2390	1967
**818**	KU7536	6 km E of Qala Shararak Ghor	Ghor	C	64.225702	34.119948	2390	1967
**819**	KU7537	6 km E of Qala Shararak Ghor	Ghor	C	64.225702	34.119948	2390	1967
**820**	KU7538	11 km E of Qala Shararak Ghor	Ghor	C	64.225702	34.119948	2490	1967
**821**	KU7539	11 km E of Qala Shararak Ghor	Ghor	C	64.225702	34.119948	2490	1967
**822**	KU7540	11 km E of Qala Shararak Ghor	Ghor	C	64.225702	34.119948	2490	1967
**823**	KU7542	16 km E of Qala Shararak Ghor	Ghor	C	64.225702	34.119948	2490	1967
**824**	KU7547	15 km SE of Dosht-i-Pam (on Tairama road) Ghor	Ghor	C	64.904251	34.081441	2640	1967
**825**	KU7550	15 km SE of Dosht-i-Pam (on Tairama road) Ghor	Ghor	C	64.904251	34.081441	2640	1967
**826**	KU7551	36 km SE of Dosht-i-Pam (on Tairama road) Ghor	Ghor	C	64.904251	34.081441	2490	1967
**827**	KU7553	36 km SE of Dosht-i-Pam (on Tairama road) Ghor	Ghor	C	64.904251	34.081441	2490	1967
**828**	KU7555	36 km SE of Dosht-i-Pam (on Tairama road) Ghor	Ghor	C	64.904251	34.081441	2490	1967
**829**	KU7556	36 km SE of Dosht-i-Pam (on Tairama road) Ghor	Ghor	C	64.904251	34.081441	2490	1967
**830**	KU7557	36 km SE of Dosht-i-Pam (on Tairama road) Ghor	Ghor	C	64.904251	34.081441	2490	1967
**831**	KU7558	60 km SE of Dosht-i-Pam (on Tairama road) Ghor	Ghor	C	64.904251	34.081441	2610	1967
**832**	KU7559	60 km SE of Dosht-i-Pam (on Tairama road) Ghor	Ghor	C	64.904251	34.081441	2610	1967
**833**	KU7560	60 km SE of Dosht-i-Pam (on Tairama road) Ghor	Ghor	C	64.904251	34.081441	2610	1967
**834**	KU7561	Tarbulock 58 km NE of Qala Shararak Ghor	Ghor	C	64.225702	34.119948	2640	1967
**835**	KU7562	Tarbulock 58 km NE of Qala Shararak Ghor	Ghor	C	64.225702	34.119948	2640	1967
**836**	KU7563	Tarbulock 58 km NE of Qala Shararak Ghor	Ghor	C	64.225702	34.119948	2640	1967
**837**	KU7564	Tarbulock 58 km NE of Qala Shararak Ghor	Ghor	C	64.225702	34.119948	2640	1967
**838**	KU7565	23 km E of Dosht-i-Pam Ghor	Ghor	C	64.904251	34.081441	2640	1967
**839**	KU7566	23 km E of Dosht-i-Pam Ghor	Ghor	C	64.904251	34.081441	2640	1967
**840**	KU7567	23 km E of Dosht-i-Pam Ghor	Ghor	C	64.904251	34.081441	2640	1967
**841**	KU7568	35 km E of Dosht-i-Pam Ghor	Ghor	C	64.904251	34.081441	2850	1967

**842**	KU7569	35 km E of Dosht-i-Pam Ghor	Ghor	C	64.904251	34.081441	2850	1967
**843**	KU7570	35 km E of Dosht-i-Pam Ghor	Ghor	C	64.904251	34.081441	2850	1967
**844**	KU7571	35 km E of Dosht-i-Pam Ghor	Ghor	C	64.904251	34.081441	2850	1967
**845**	KU7572	46 km NE of Dosht-i-Pam Ghor	Ghor	C	64.904251	34.081441	2640	1967
**846**	KU7573	46 km NE of Dosht-i-Pam Ghor	Ghor	C	64.904251	34.081441	2640	1967
**847**	KU7574	46 km NE of Dosht-i-Pam Ghor	Ghor	C	64.904251	34.081441	2640	1967
**848**	KU7575	46 km NE of Dosht-i-Pam Ghor	Ghor	C	64.904251	34.081441	2640	1967
**849**	KU7576	3 km W of Qala Ahangharan Ghor	Ghor	C	65.126037	34.322116	2120	1967
**850**	KU7577	3 km W of Qala Ahangharan Ghor	Ghor	C	65.126037	34.322116	2120	1967
**851**	KU7578	3 km W of Qala Ahangharan Ghor	Ghor	C	65.126037	34.322116	2120	1967
**852**	KU7579	3 km W of Qala Ahangharan Ghor	Ghor	C	65.126037	34.322116	2120	1967
**853**	KU7580	11 km W of Qala Ahangharan Ghor	Ghor	C	65.126037	34.322116	2180	1967
**854**	KU7582	11 km W of Qala Ahangharan Ghor	Ghor	C	65.126037	34.322116	2180	1967
**855**	KU7583	15 km W of Qala Ahangharan Ghor	Ghor	C	65.126037	34.322116	2180	1967
**856**	KU7584	--	unknown	unknown	69.171181	34.532751	-	1967
**857**	KU7585	1.6 km W of Qala Ahangharan Ghor	Ghor	C	65.126037	34.322116	2240	1967
**858**	KU7586	1.6 km W of Qala Ahangharan Ghor	Ghor	C	65.126037	34.322116	2240	1967
**859**	KU7587	11 km S of Kilmin Ghor	Ghor	C	64.92897	34.10646	2580	1967
**860**	KU7589	11 km S of Kilmin Ghor	Ghor	C	64.92897	34.10646	2580	1967
**861**	KU7591	--	unknown	unknown	69.171181	34.532751	-	1967
**862**	KU7592	Kilmin Ghor	Ghor	C	64.92897	34.10646	2090	1967
**863**	KU7593	Kilmin Ghor	Ghor	C	64.92897	34.10646	2090	1967
**864**	KU7595	Kilmin Ghor	Ghor	C	64.92897	34.10646	2090	1967
**865**	KU7596	20 km E of Qala Ahangharan Ghor	Ghor	C	65.126037	34.322116	2300	1967
**866**	KU7597	20 km E of Qala Ahangharan Ghor	Ghor	C	65.126037	34.322116	2300	1967
**867**	KU7598	15 km E of Badgah Ghor	Ghor	C	65.424342	34.514924	2420	1967
**868**	KU7599	15 km E of Badgah Ghor	Ghor	C	65.424342	34.514924	2420	1967
**869**	KU7600	15 km E of Badgah Ghor	Ghor	C	65.424342	34.514924	2420	1967
**870**	KU7602	Daulat Yar Ghor	Ghor	C	64.845886	34.113169	2460	1967
**871**	KU7604	40 km N of Herat Herat	Herat	C	62.082825	34.285815	1490	1967
**872**	KU7605	Gormos village Ghor	Ghor	C	64.845886	34.113169	2760	1967
**873**	KU7606	15 km E of Qizil Ghor	Ghor	C	66.020164	34.459183	2880	1967
**874**	KU7607	15 km E of Qizil Ghor	Ghor	C	66.020164	34.459183	2880	1967
**875**	KU7608	30 km E of Qizil Ghor	Ghor	C	66.020164	34.459183	2790	1967
**876**	KU7609	3 km E of Qala Sakawa (Daulat Yar - Panjao) Ghor	Ghor	C	66.274717	34.498751	2790	1967
**877**	KU7610	3 km E of Qala Sakawa (Daulat Yar - Panjao) Ghor	Ghor	C	66.274717	34.498751	2790	1967
**878**	KU7611	3 km E of Qala Sakawa (Daulat Yar - Panjao) Ghor	Ghor	C	66.274717	34.498751	2790	1967

879	KU7612	3 km E of Qala Sakawa (Daulat Yar - Panjao) Ghor	Ghor	C	66.274717	34.498751	2790	1967
880	KU7613	33 km E of Qala Sakawa (Daulat Yar - Panjao) Ghor	Ghor	C	66.274717	34.498751	3000	1967
881	KU7614	5 km W of Banki (Daulat Yar - Panjao) Ghor	Ghor	C	64.402428	33.45224	2910	1967
882	KU7615	5 km W of Banki (Daulat Yar - Panjao) Ghor	Ghor	C	64.402428	33.45224	2910	1967
883	KU7616	15 km W of Panjao Bamiyan	Bamyan	E	67.006874	34.417843	2910	1967
884	KU7617	21 km N of Panjao Bamiyan	Bamyan	E	67.006874	34.417843	3030	1967
885	KU7618	6.6 km N of Panjao Bamiyan	Bamyan	E	67.006874	34.417843	2790	1967
886	KU7619	3 km S of Naik (Panjao - Bamian) Bamiyan	Bamyan	E	67.00676	34.744208	2730	1967
887	KU7620	11 km E of Naik (Panjao - Bamian) Bamiyan	Bamyan	E	67.00676	34.744208	2700	1967
888	KU7621	11 km E of Naik (Panjao - Bamian) Bamiyan	Bamyan	E	67.00676	34.744208	2700	1967
889	KU7622	16 km E of Naik (Panjao - Bamian) Bamiyan	Bamyan	E	67.00676	34.744208	2940	1967
890	KU7623	Shinbatu (Panjao - Bamian) Bamiyan	Bamyan	E	68.021393	34.940661	3090	1967
891	KU7624	15 km W of Bamian (Panjao - Bamian) Bamiyan	Bamyan	E	67.790909	34.841197	2640	1967
892	KU7626	Bamian (Panjao - Bamian) Bamiyan	Bamyan	E	67.790909	34.841197	2580	1967
893	KU7627	6.6 km E of Bamian Bamiyan	Bamyan	E	67.790909	34.841197	2520	1967
894	KU7630	1.6 km E of Ghorband (E of Bamian) Parwan	Parwan	E	66.455383	35.067322	2000	1967
895	KU7632	1.6 km E of Ghorband (E of Bamian) Parwan	Parwan	E	66.455383	35.067322	2000	1967
896	KU7634	near Shutue Shahe village Parwan	Parwan	E	68.957977	35.185706	1820	1967
897	KU7637	5 km E of Unai Pass Wardak	Wardak	F	68.376102	34.450605	2850	1967
898	KU7638	5 km E of Unai Pass Wardak	Wardak	F	68.376102	34.450605	2850	1967
899	KU7640	Unai Pass - below peak Wardak	Wardak	F	68.376102	34.450605	3000	1967
900	KU7641	Unai Pass - below peak Wardak	Wardak	F	68.376102	34.450605	3000	1967
901	KU7642	Unai (below Pass) Wardak	Wardak	F	68.376102	34.450605	3000	1967
902	KU7643	110 km N of Kabul - Salang Pass (Doshi - Charikar) Baghlan	Baghlan	B	68.690414	36.236852	2240	1967
903	KU7644	Taliqan Takar	Takhar	B	69.166259	34.566288	850	1967
904	KU7646	5 km W of Mazar-i-Sharif Balkh	Balkh	A	67.109613	36.703288	420	1967
905	KU7647	5 km W of Mazar-i-Sharif Balkh	Balkh	A	67.109613	36.703288	420	1967
906	KU7648	25 km S of Tash-Kurghan near Khulm Samangan	Samangan	A	67.693105	36.687026	790	1967
907	KU7649	3 km S of Daulatabad Balkh	Balkh	A	66.814982	36.98874	390	1967
908	KU7650	3 km S of Daulatabad Balkh	Balkh	A	66.814982	36.98874	390	1967
909	KU7651	3 km S of Daulatabad Balkh	Balkh	A	66.814982	36.98874	390	1967
910	KU7652	3 km S of Daulatabad Balkh	Balkh	A	66.814982	36.98874	390	1967
911	KU7653	5 km W of Tash-Kurghan near Khulm Samangan	Samangan	A	67.693105	36.687026	490	1967
912	KU7654	13 km S of Tash-Kurghan near Khulm Samangan	Samangan	A	67.693105	36.687026	670	1967
913	KU7655	13 km S of Tash-Kurghan near Khulm Samangan	Samangan	A	67.693105	36.687026	670	1967
914	KU7656	13 km S of Tash-Kurghan near Khulm Samangan	Samangan	A	67.693105	36.687026	670	1967
915	KU7657	25 km S of Tash-Kurghan near Khulm Samangan	Samangan	A	67.693105	36.687026	790	1967

**916**	KU7658	5 km SW of Haibak Samangan	Samangan	A	68.026886	36.240507	1030	1967
**917**	KU7659	5 km SW of Haibak Samangan	Samangan	A	68.026886	36.240507	1030	1967
**918**	KU7660	16 km SW of Haibak Samangan	Samangan	A	68.026886	36.240507	1090	1967
**919**	KU7661	16 km SW of Haibak Samangan	Samangan	A	68.026886	36.240507	1090	1967
**921**	KU7663	8 km S of Salang Pass (Doshi - Charikar) Baghlan	Baghlan	B	68.689041	35.837076	2670	1967
**922**	KU7664	43 km SW of Ghazni Ghazni	Ghazni	D	68.378448	33.535214	2090	1967
**923**	KU7665	43 km SW of Ghazni Ghazni	Ghazni	D	68.378448	33.535214	2090	1967
**924**	KU7666	105 km SW of Ghazni Ghazni	Ghazni	D	68.378448	33.535214	2070	1967
**925**	KU7667	93 km NE of Kandahar Kandahar	Kandahar	H	65.756207	31.599387	1390	1967
**926**	KU7668	45 km NE of Kandahar Kandahar	Kandahar	H	65.756207	31.599387	1210	1967
**927**	KU7669	16 km W of Kandahar Kandahar	Kandahar	H	65.756207	31.599387	1000	1967
**928**	KU7670	Daulat Yar Ghor	Ghor	C	64.845886	34.113169	2460	1967
**929**	KU7671	75 km W of Kandahar Kandahar	Kandahar	H	65.756207	31.599387	1000	1967
**930**	KU7672	6.6 km S of Kandahar Kandahar	Kandahar	H	65.756207	31.599387	850	1967
**931**	KU7673	--	unknown	unknown	69.171181	34.532751	-	1967
**932**	KU7675	--	unknown	unknown	69.171181	34.532751	-	1967
**933**	KU7676	--	unknown	unknown	69.171181	34.532751	-	1967
**934**	KU7678	--	unknown	unknown	69.171181	34.532751	-	1967
**935**	KU7679	--	unknown	unknown	69.171181	34.532751	-	1967
**936**	KU7681	--	unknown	unknown	69.171181	34.532751	-	1967
**937**	KU7682A	--	unknown	unknown	69.171181	34.532751	-	1967
**938**	KU7682B	--	unknown	unknown	69.171181	34.532751	-	1967
**939**	KU7683	--	unknown	unknown	69.171181	34.532751	-	1967
**940**	KU7684	--	unknown	unknown	69.171181	34.532751	-	1967
**941**	KU7686	--	unknown	unknown	69.171181	34.532751	-	1967
**942**	KU7689	--	unknown	unknown	69.171181	34.532751	-	1967
**943**	KU7690	--	unknown	unknown	69.171181	34.532751	-	1967
**944**	KU11239B	Puli-zeribon Shewa Badakhshan	Badakhshan	B	70.488281	33.266824	2540	1979
**955**	KU1824	Pagman	Kabul	F	68.875236	34.64976	-	1967
**956**	KU1825	Pagman	Kabul	F	68.875236	34.64976	-	1967
**958**	KU7431	1.5 km E of Obeh (1.7 km N of Hari rud River) Herat	Herat	C	65.257873	32.274593	1360	1967
**960**	KU7439	4 km E of Armalik Herat	Herat	C	62.190113	34.376681	1550	1967
**961**	KU7440	4 km E of Armalik Herat	Herat	C	62.190113	34.376681	1550	1967
**968**	KU7500	28 km E of Besha Herat	Herat	C	69.153385	34.540868	1580	1967
**975**	KU7590	11 km S of Kilmin Ghor	Ghor	C	64.92897	34.10646	2580	1967
**977**	KU7629	66 km W of Ghorband (E of Bamian) Parwan	Herat	C	69.455337	34.139429	2610	1967
**979**	KU7639	5 km E of Unai Pass Wardak	Wardak	F	68.376102	34.450605	2850	1967

981	KU7674	--	unknown	unknown	69.171181	34.532751	-	1967
		**NBRP stands for National Bio-Resource Project, Japan**						
