# Supplementary figures and images for: Molecular evaluation of orphan Afghan common wheat (Triticum aestivum L.) landraces collected by Dr. Kihara using single nucleotide polymorphic markers
Source: BMC Plant Biol. 2014 Nov 29;14:320. doi: 10.1186/s12870-014-0320-5 (PMC4255927; doi:10.1186/s12870-014-0320-5)

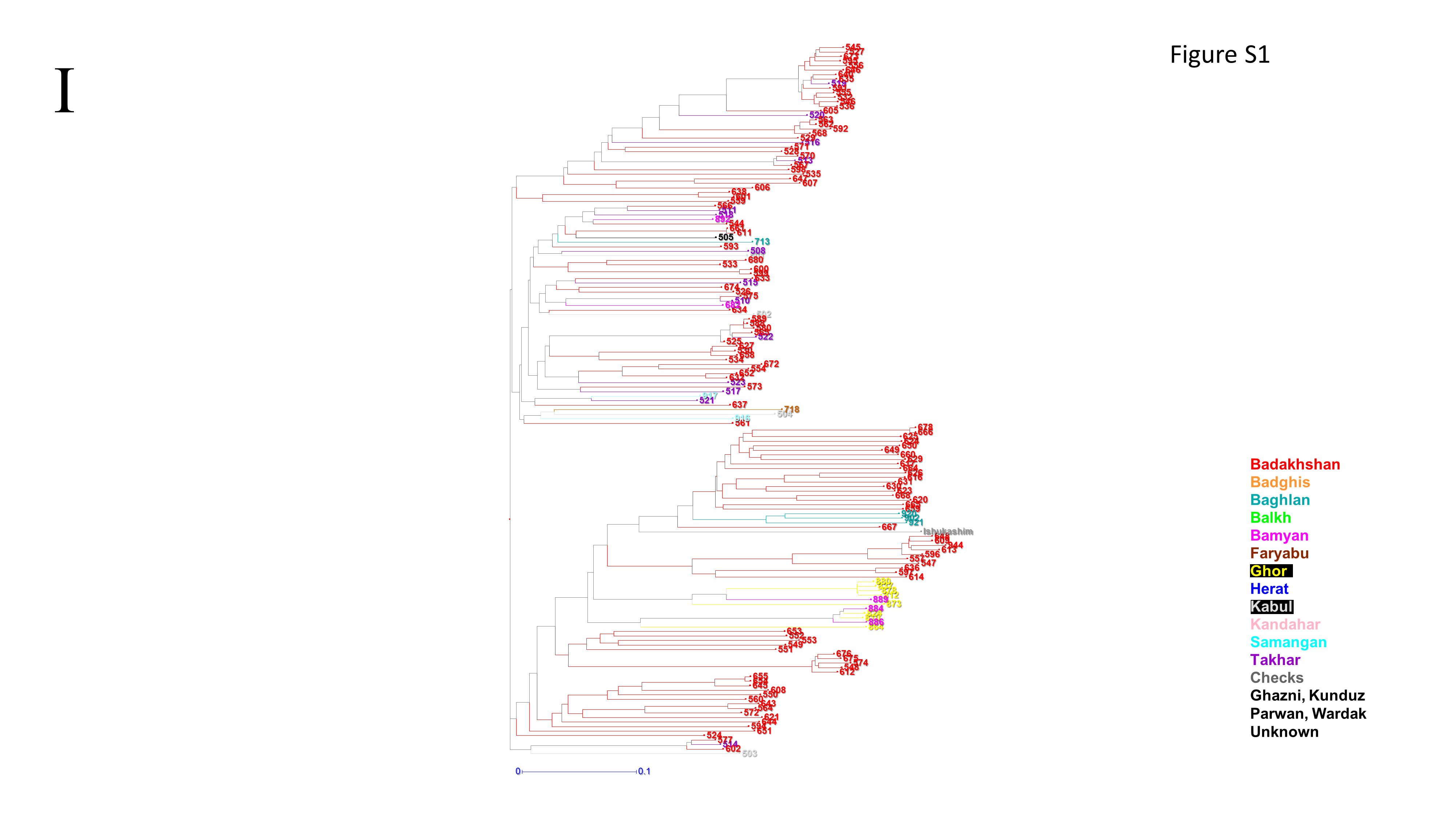

Supplement: Additional file 2: — Dendrograms for each clade of the landrace germplasm. [file 12870_2014_320_MOESM2_ESM.zip › Slide1.TIF]

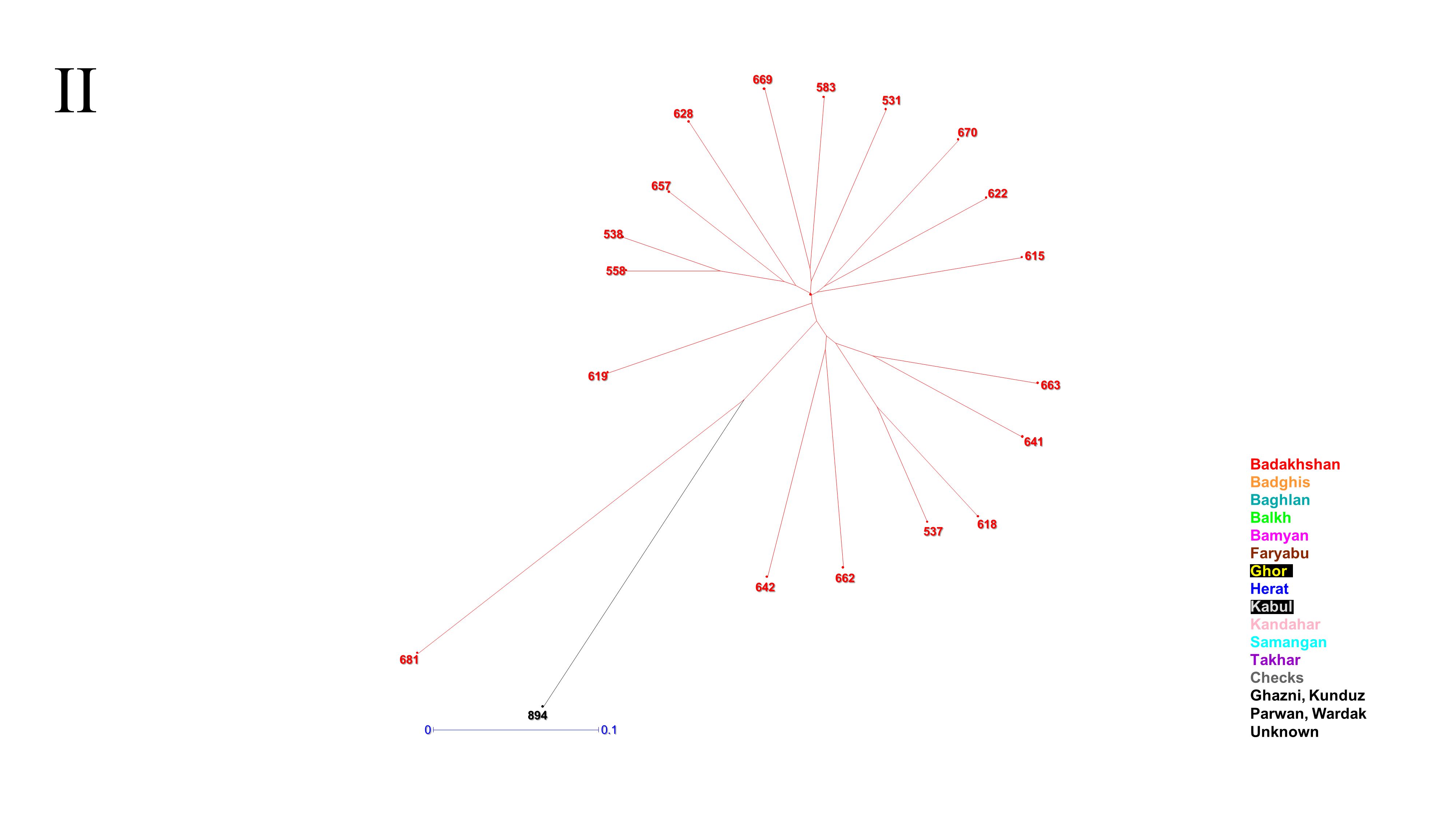

Supplement: Additional file 2: — Dendrograms for each clade of the landrace germplasm. [file 12870_2014_320_MOESM2_ESM.zip › Slide2.TIF]

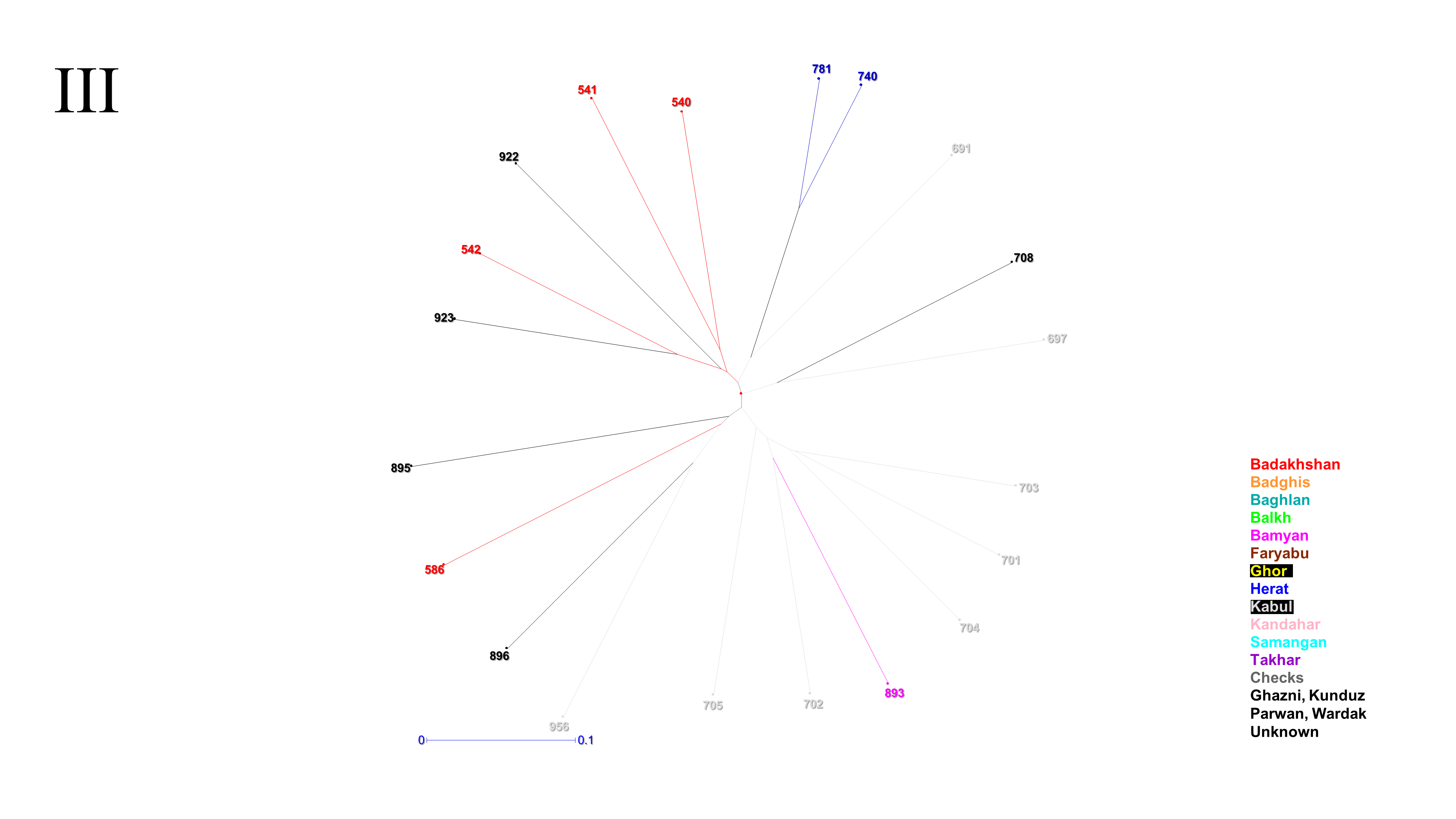

Supplement: Additional file 2: — Dendrograms for each clade of the landrace germplasm. [file 12870_2014_320_MOESM2_ESM.zip › Slide3.TIF]

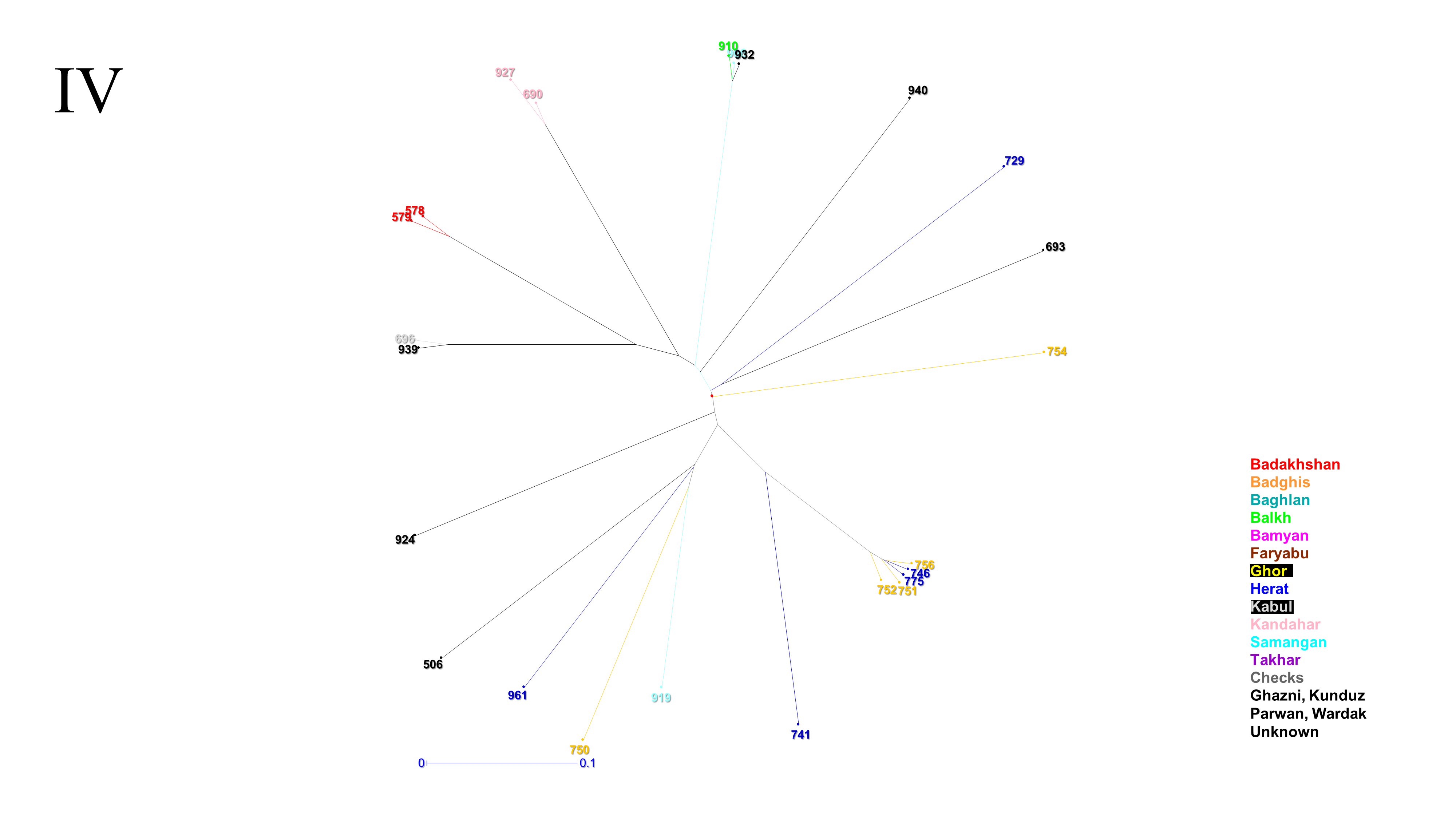

Supplement: Additional file 2: — Dendrograms for each clade of the landrace germplasm. [file 12870_2014_320_MOESM2_ESM.zip › Slide4.TIF]

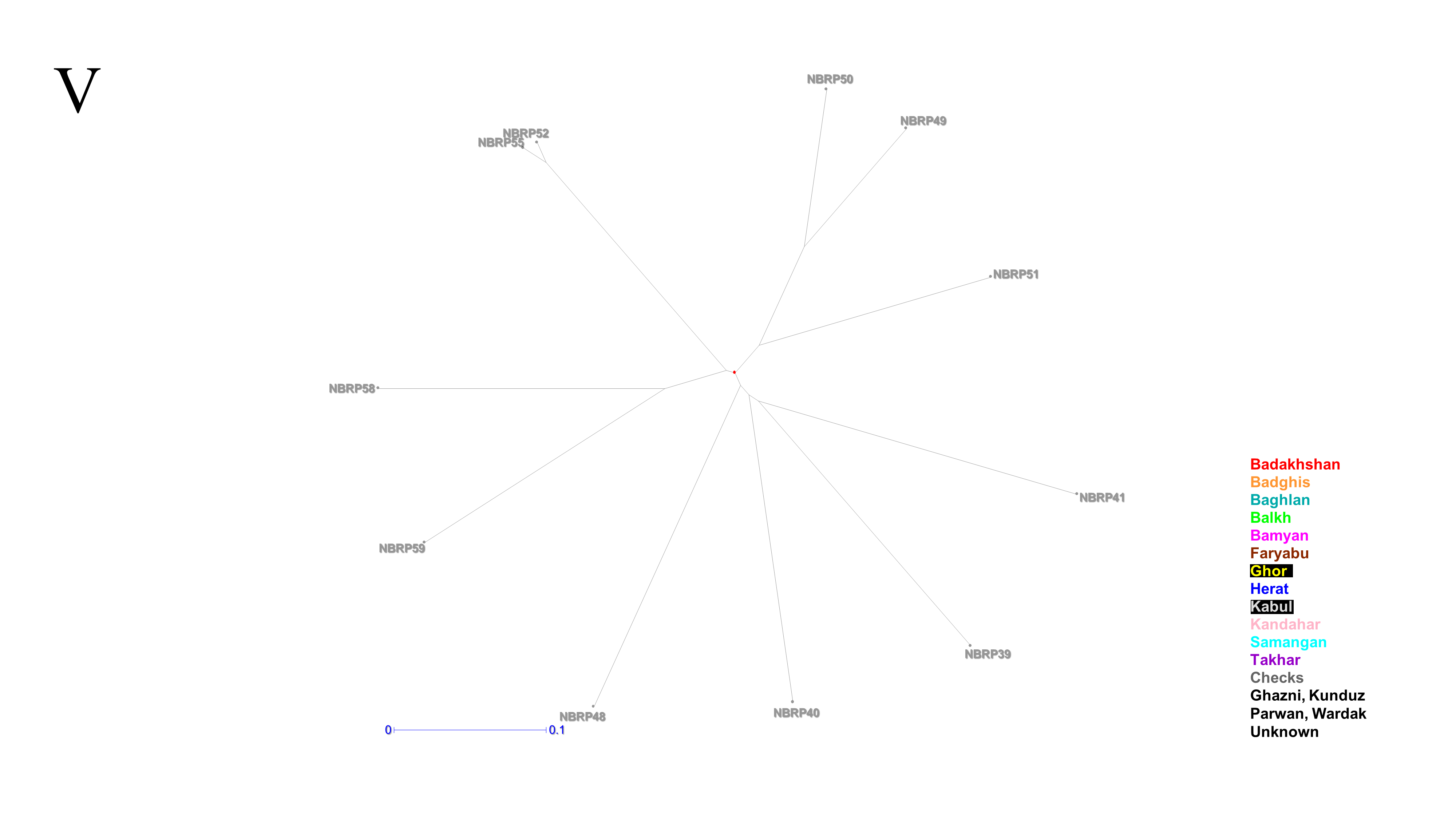

Supplement: Additional file 2: — Dendrograms for each clade of the landrace germplasm. [file 12870_2014_320_MOESM2_ESM.zip › Slide5.TIF]

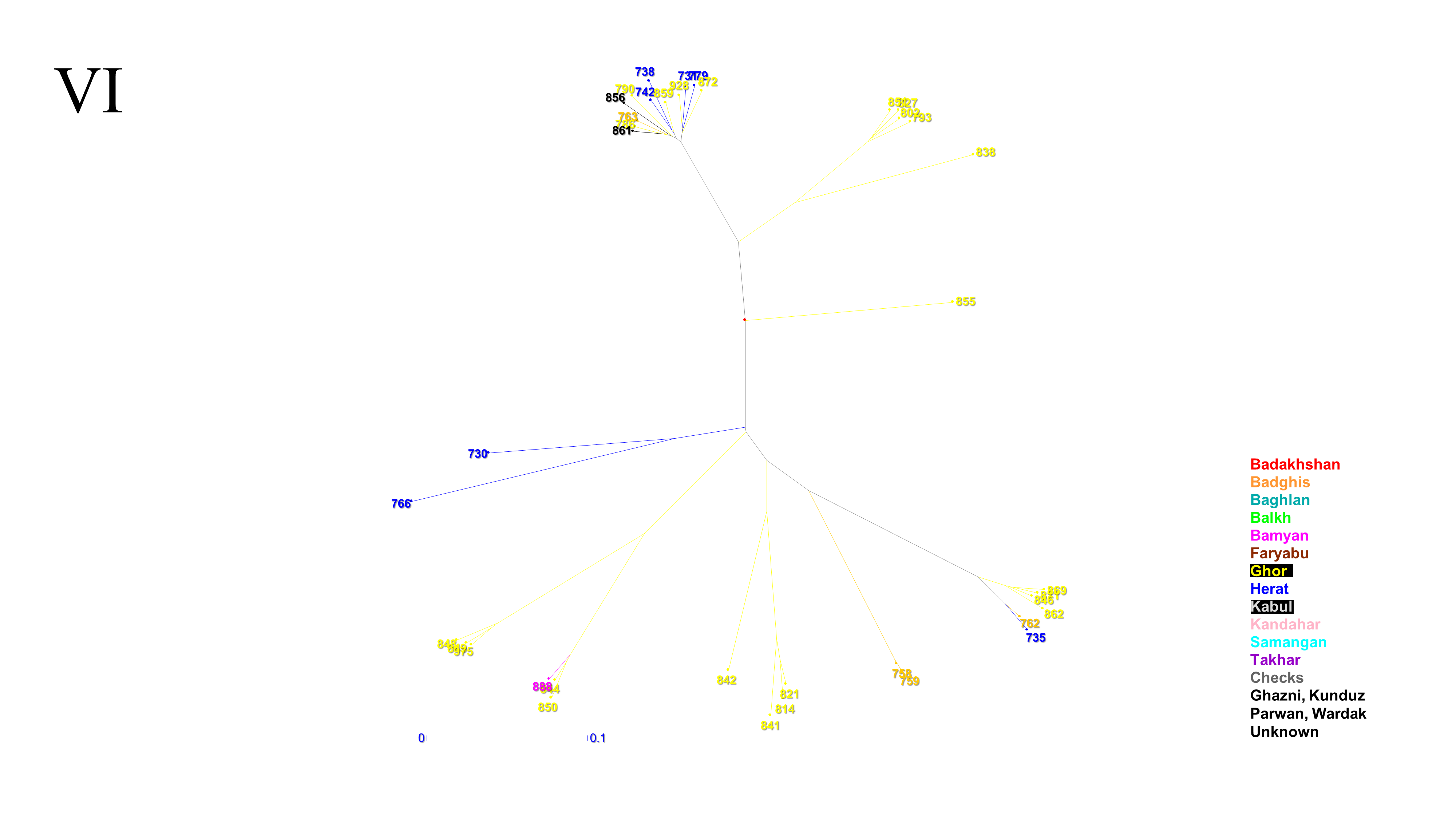

Supplement: Additional file 2: — Dendrograms for each clade of the landrace germplasm. [file 12870_2014_320_MOESM2_ESM.zip › Slide6.TIF]

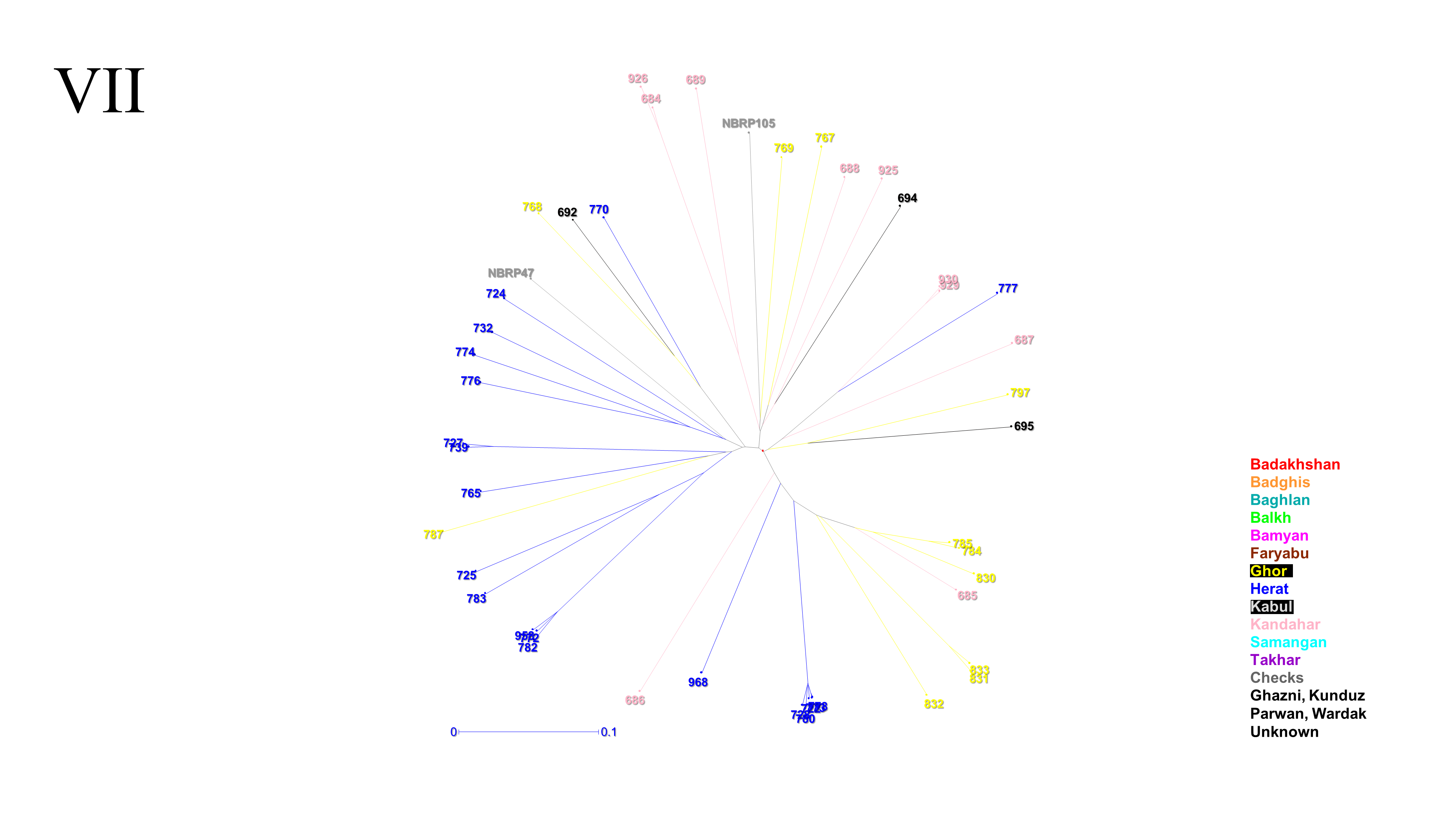

Supplement: Additional file 2: — Dendrograms for each clade of the landrace germplasm. [file 12870_2014_320_MOESM2_ESM.zip › Slide7.TIF]

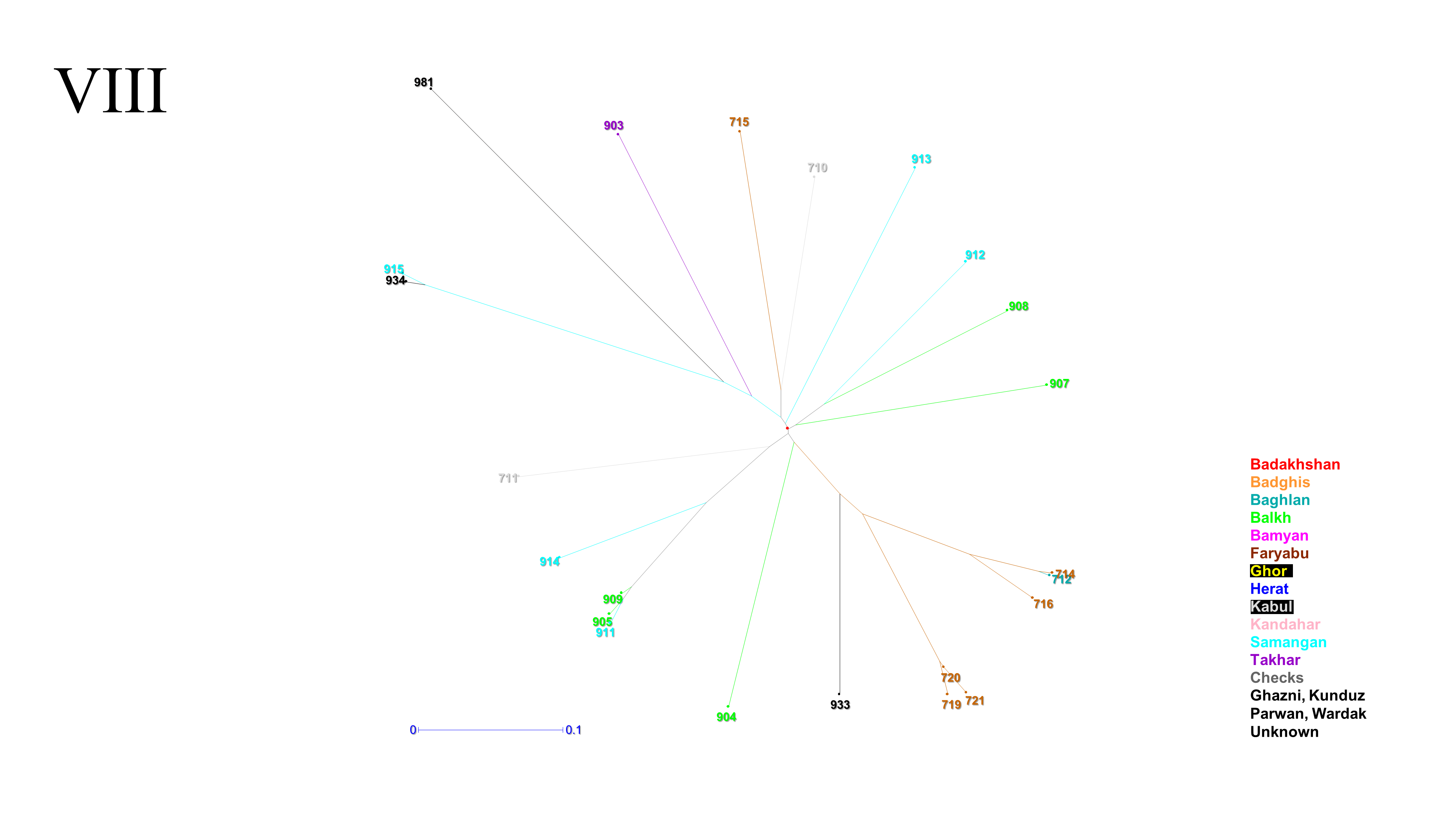

Supplement: Additional file 2: — Dendrograms for each clade of the landrace germplasm. [file 12870_2014_320_MOESM2_ESM.zip › Slide8.TIF]

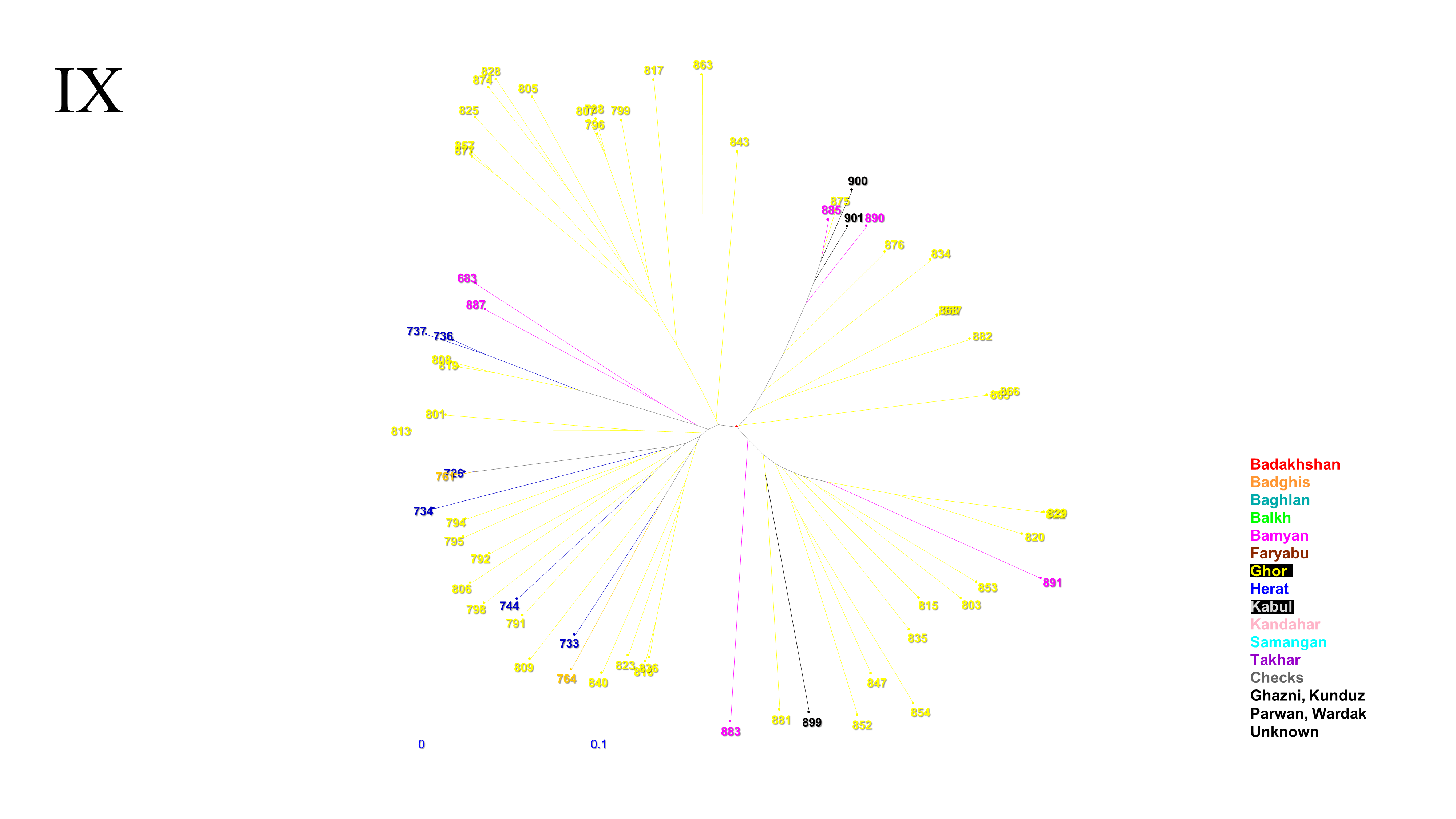

Supplement: Additional file 2: — Dendrograms for each clade of the landrace germplasm. [file 12870_2014_320_MOESM2_ESM.zip › Slide9.TIF]

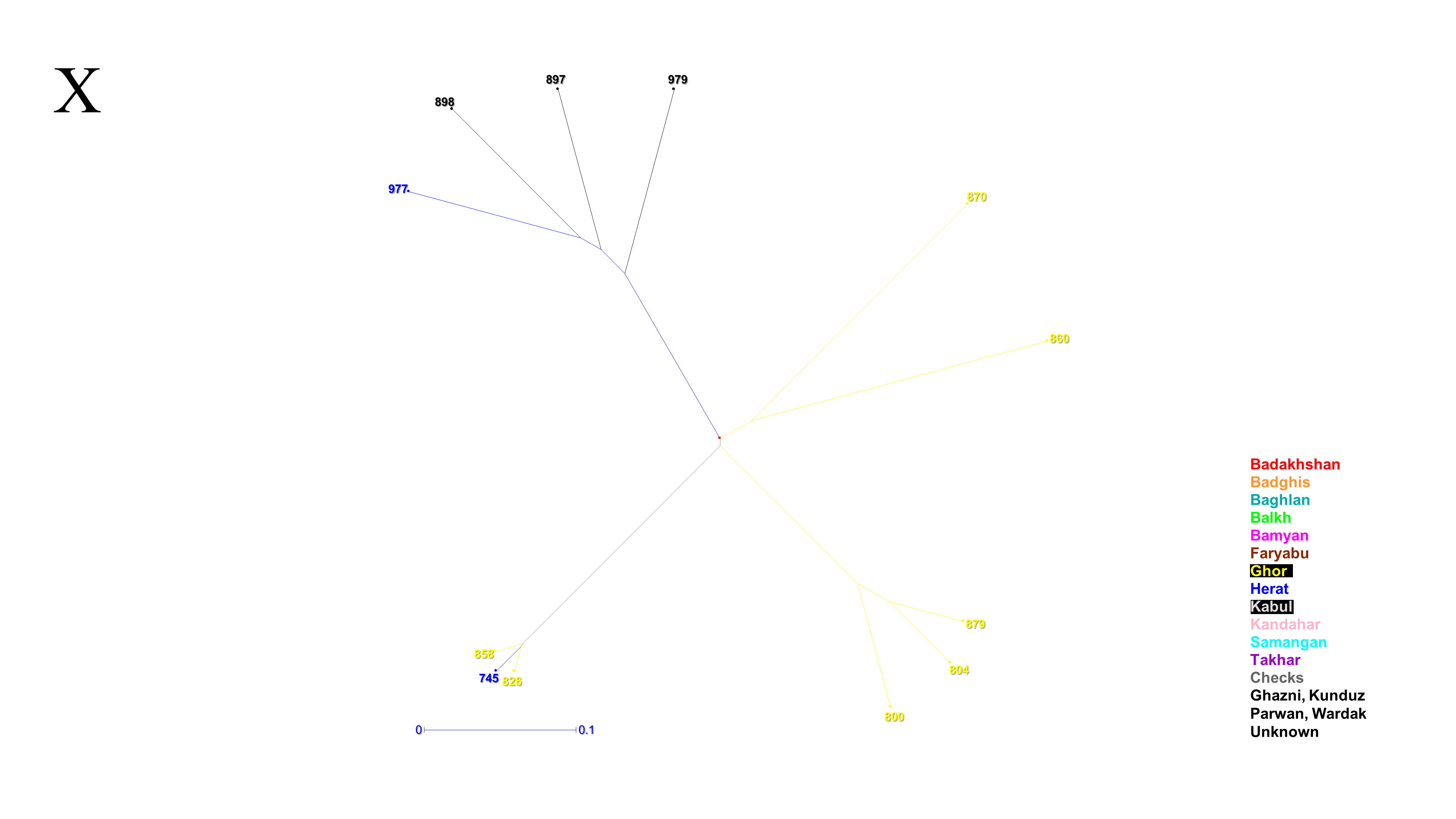

Supplement: Additional file 2: — Dendrograms for each clade of the landrace germplasm. [file 12870_2014_320_MOESM2_ESM.zip › Slide10.TIF]

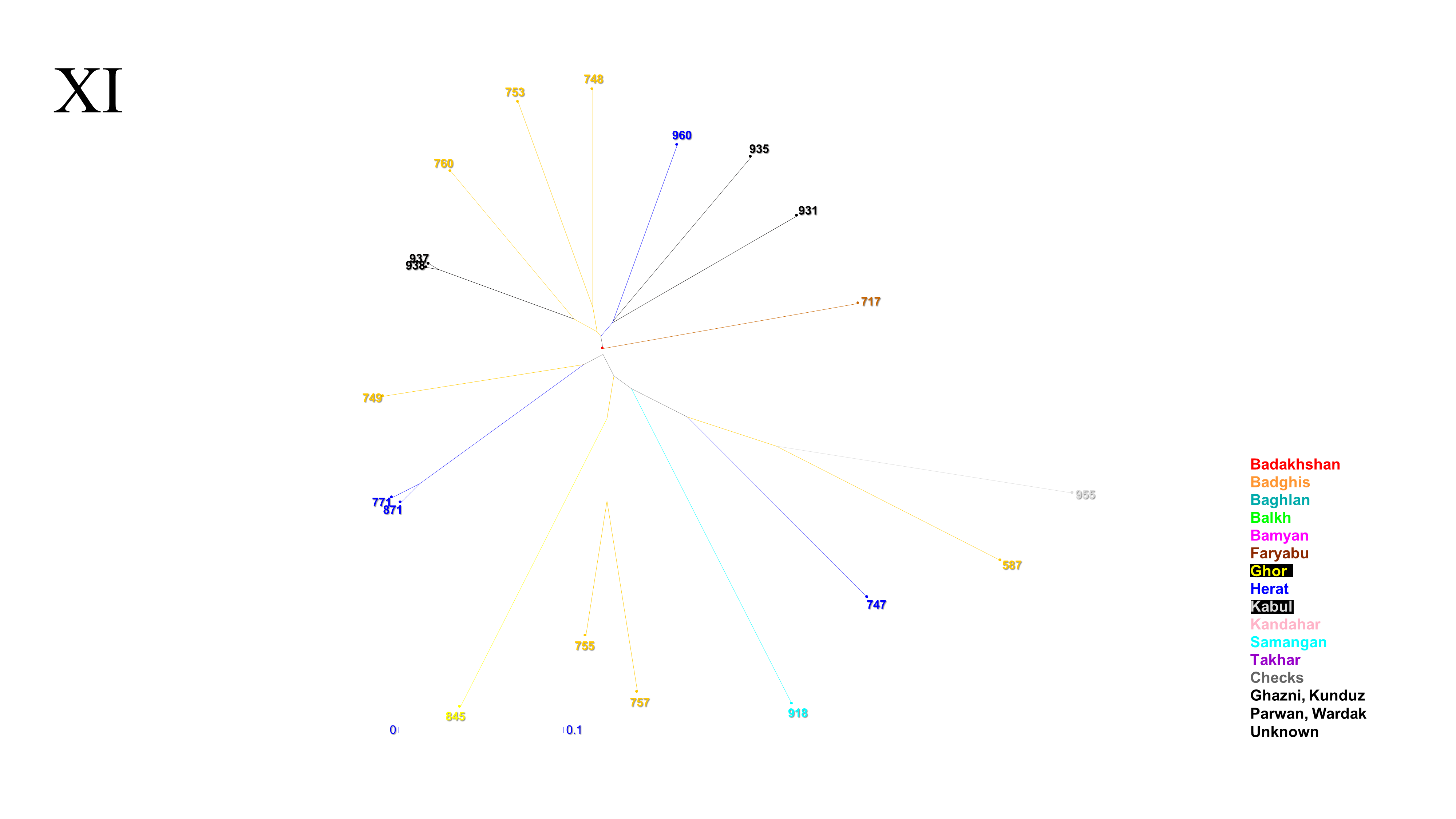

Supplement: Additional file 2: — Dendrograms for each clade of the landrace germplasm. [file 12870_2014_320_MOESM2_ESM.zip › Slide11.TIF]

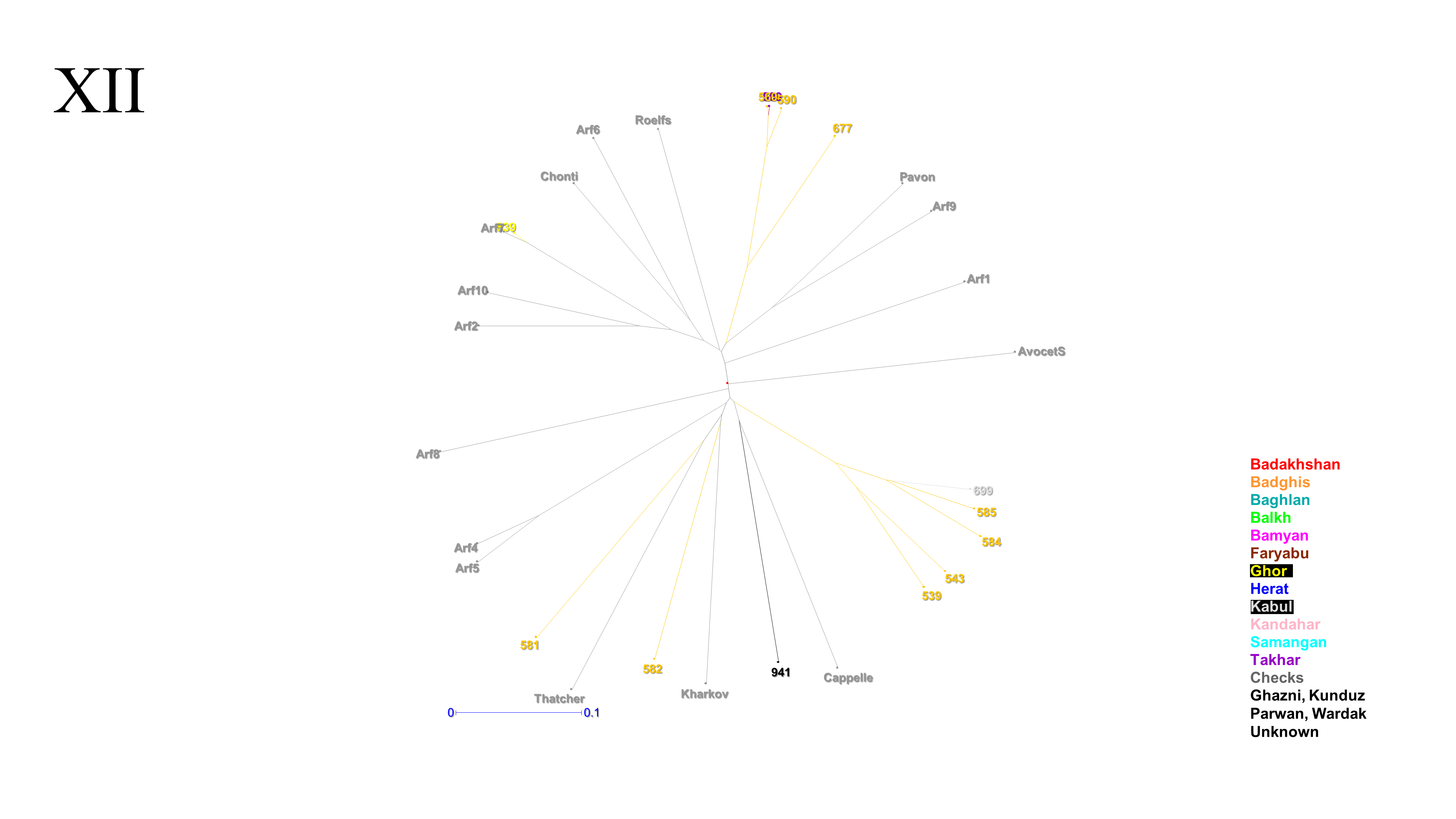

Supplement: Additional file 2: — Dendrograms for each clade of the landrace germplasm. [file 12870_2014_320_MOESM2_ESM.zip › Slide12.TIF]

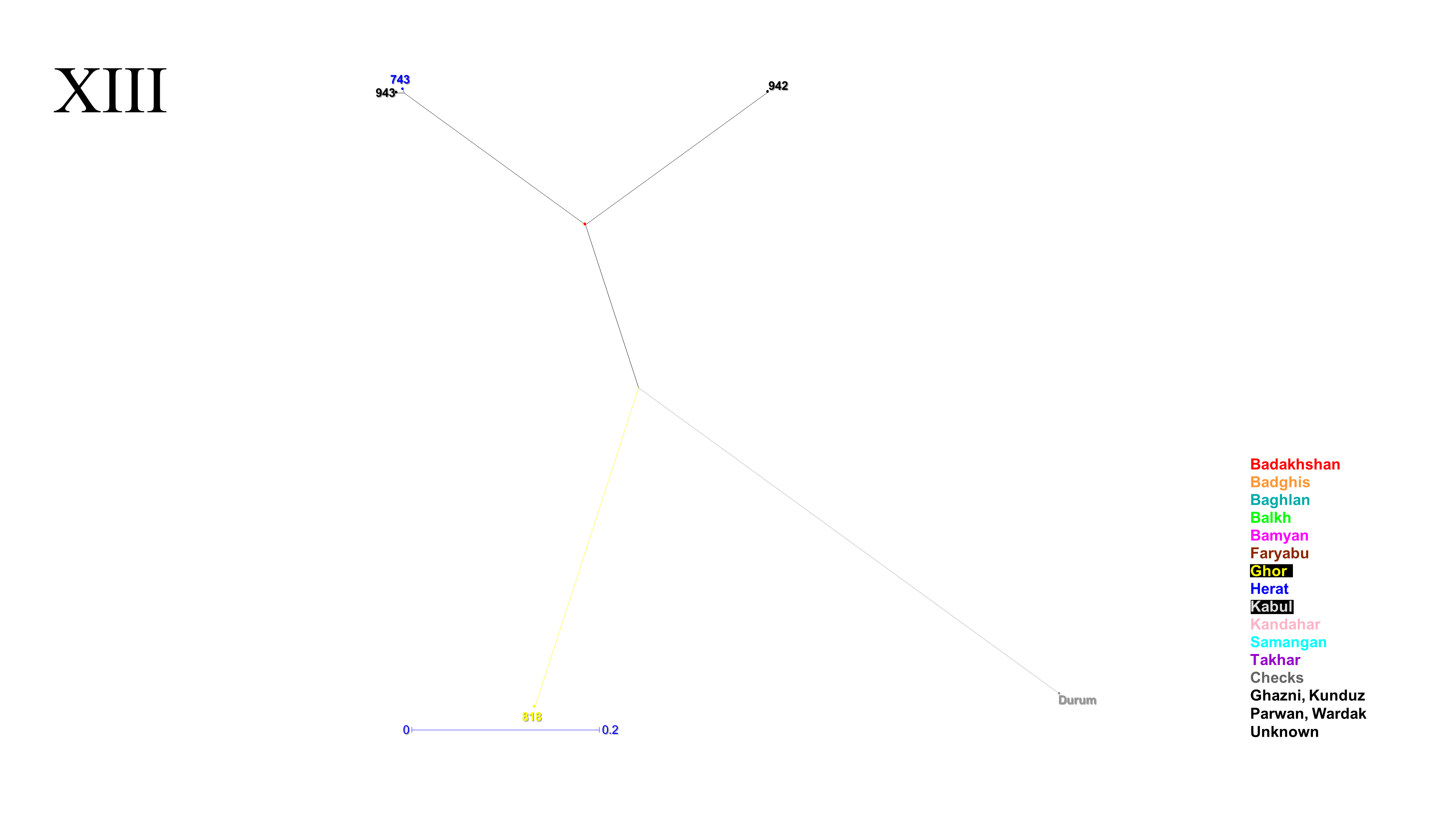

Supplement: Additional file 2: — Dendrograms for each clade of the landrace germplasm. [file 12870_2014_320_MOESM2_ESM.zip › Slide13.TIF]

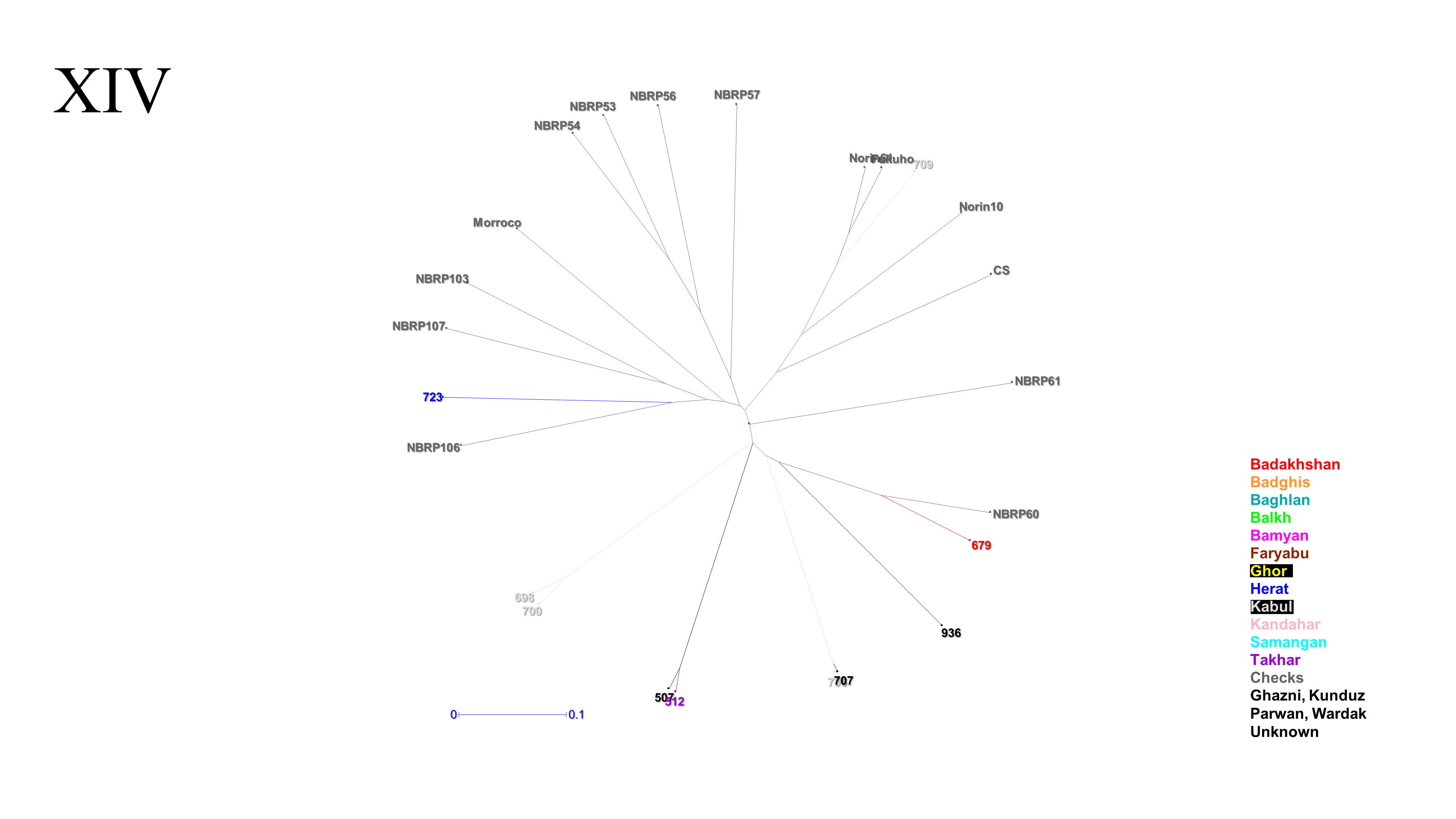

Supplement: Additional file 2: — Dendrograms for each clade of the landrace germplasm. [file 12870_2014_320_MOESM2_ESM.zip › Slide14.TIF]
