# Supplementary material for: Molecular evaluation of orphan Afghan common wheat (Triticum aestivum L.) landraces collected by Dr. Kihara using single nucleotide polymorphic markers
Source: BMC Plant Biol. 2014 Nov 29;14:320. doi: 10.1186/s12870-014-0320-5 (PMC4255927; doi:10.1186/s12870-014-0320-5)
Supplement: Additional file 3: — The control varieties used in this study. NBRP; National Bio-Resource Project, Japan. [file 12870_2014_320_MOESM3_ESM.pdf]

Table S2. Details of check varieties used in this study

Origin of the germplasm	Number of Accessions	Name of variety / cultivar
Afghan modern cultivars	11	Bakhtawar, Lalmi 2, Rana 96, Darulaman 7, Ariana 07, Chonti, Herat 99, Mazar 99, Parva 2, Kabul 2000, Solh 02
CIMMYT varieties	4	Pavon, Roelf, Cappelle, Avocets, Morocco
Land races from Pakistan	5	NBRP39, NBRP40, NBRP41, NBRP60 and NBRP61
Land races from Iran	17	NBRP47, NBRP48, NBRP49, NBRP50, NBRP51, NBRP52, NBRP53, NBRP54, NBRP55, NBRP56, NBRP57, NBRP58, NBRP59, NBRP103, NBRP105, NBRP106 and NBRP107
Japanese cultivars	4	Fukuho, Norin 61, Norin 10 and Nobeokabouzo-komugi
Others	6	Chinese spring, Sumai 3, Synthetic wheat line (ABD4), *Aegilops tauschii,* Kharkov

NBRP stands for National Bio-Resource Project, Japan
